# Bidirectional epithelial–mesenchymal crosstalk provides self-sustaining profibrotic signals in pulmonary fibrosis

**DOI:** 10.1016/j.jbc.2021.101096

**Published:** 2021-08-18

**Authors:** Liudi Yao, Yilu Zhou, Juanjuan Li, Leanne Wickens, Franco Conforti, Anna Rattu, Fathima Maneesha Ibrahim, Aiman Alzetani, Ben G. Marshall, Sophie V. Fletcher, David Hancock, Tim Wallis, Julian Downward, Rob M. Ewing, Luca Richeldi, Paul Skipp, Donna E. Davies, Mark G. Jones, Yihua Wang

**Affiliations:** 1 Biological Sciences, Faculty of Environmental and Life Sciences, University of Southampton, Southampton, United Kingdom; 2 Institute for Life Sciences, University of Southampton, Southampton, United Kingdom; 3 Centre for Proteomic Research, Institute for Life Sciences, University of Southampton, Southampton, United Kingdom; 4 Clinical and Experimental Sciences, Faculty of Medicine, University of Southampton, Southampton, United Kingdom; 5 NIHR Southampton Biomedical Research Centre, University Hospital Southampton, Southampton, United Kingdom; 6 University Hospital Southampton, Southampton, United Kingdom; 7 Oncogene Biology, The Francis Crick Institute, London, United Kingdom; 8 Unità Operativa Complessa di Pneumologia, Università Cattolica del Sacro Cuore, Fondazione Policlinico A. Gemelli, Rome, Italy

**Keywords:** pulmonary fibrosis, epithelial–mesenchymal transition, ZEB1, SPARC, EGFR, RAS, TGF-β, ATII, alveolar epithelial type II, CM, conditioned media, DEG, differentially expressed gene, ECM, extracellular matrix, EGFR, epithelial growth factor receptor, EMT, epithelial–mesenchymal transition, ERK, extracellular-regulated kinase, FDR, false discovery rate, GO, Gene Ontology, GSVA, gene set variation analysis, IPF, idiopathic pulmonary fibrosis, IPFF, IPF fibroblast, NHLF, normal human lung fibroblast, 4-OHT, 4-hydroxytamoxifen, α-SMA, α-smooth muscle actin, SPARC, secreted protein acidic and rich in cysteine, TGF-β, transforming growth factor-β, tPA, tissue plasminogen activator, ZEB1, zinc finger E-box-binding homeobox 1

## Abstract

Idiopathic pulmonary fibrosis (IPF) is the prototypic progressive fibrotic lung disease with a median survival of 2 to 4 years. Injury to and/or dysfunction of the alveolar epithelium is strongly implicated in IPF disease initiation, but the factors that determine whether fibrosis progresses rather than normal tissue repair occurs remain poorly understood. We previously demonstrated that zinc finger E-box-binding homeobox 1–mediated epithelial–mesenchymal transition in human alveolar epithelial type II (ATII) cells augments transforming growth factor-β–induced profibrogenic responses in underlying lung fibroblasts *via* paracrine signaling. Here, we investigated bidirectional epithelial–mesenchymal crosstalk and its potential to drive fibrosis progression. RNA-Seq of lung fibroblasts exposed to conditioned media from ATII cells undergoing RAS-induced epithelial–mesenchymal transition identified many differentially expressed genes including those involved in cell migration and extracellular matrix regulation. We confirmed that paracrine signaling between RAS-activated ATII cells and fibroblasts augmented fibroblast recruitment and demonstrated that this involved a zinc finger E-box-binding homeobox 1–tissue plasminogen activator axis. In a reciprocal fashion, paracrine signaling from transforming growth factor-β–activated lung fibroblasts or IPF fibroblasts induced RAS activation in ATII cells, at least partially through the secreted protein acidic and rich in cysteine, which may signal *via* the epithelial growth factor receptor *via* epithelial growth factor–like repeats. Together, these data identify that aberrant bidirectional epithelial–mesenchymal crosstalk in IPF drives a chronic feedback loop that maintains a wound-healing phenotype and provides self-sustaining profibrotic signals.

Fibrotic diseases are a major cause of morbidity and mortality worldwide, and their prevalence is increasing with an ageing population (1). Development of effective treatments for progressive fibrosis is considered one of the most challenging tasks in modern medicine.

Our studies focus within the lung upon fibrotic interstitial lung diseases, a diverse group of conditions that affect the space between the alveolar epithelium and the capillary endothelium to cause varying degrees of inflammation and fibrosis. Once considered rare, epidemiologic investigations have found them to be more common than previously recognized, with interstitial lung abnormalities detected in 7% of the general population (aged older than 50 years) resulting in an increase in all-cause mortality (hazard ratio = 2.7) (2). Idiopathic pulmonary fibrosis (IPF) is the prototypic chronic progressive fibrotic interstitial lung disease, affecting five million people worldwide (3). Like many fibrotic disorders, IPF is characterized by enhanced deposition and remodeling of the extracellular matrix (ECM). This leads to decreased lung compliance, disrupted gas exchange, and ultimately respiratory failure and death. Median survival from diagnosis is 2 to 4 years, and approved therapies only slow disease progression, so there is a significant unmet need.

An overexuberant wound healing response is regarded as a canonical cause of organ fibrosis (4). Abnormal wound healing may occur as a consequence of multiple stimuli, including infections, chemical exposures, and physical injuries (1, 5). Whilst injury to and/or dysfunction of the alveolar epithelium is strongly implicated in IPF disease initiation, the factors that determine whether fibrosis progresses rather than normal tissue repair occurs remain poorly understood.

In this study, we identify that aberrant bidirectional epithelial–mesenchymal crosstalk provides self-sustaining activation signals driving disease progression in pulmonary fibrosis. Building on our previous report (6), we provide evidence that alveolar epithelial type II (ATII) cells undergoing RAS-induced epithelial–mesenchymal transition (EMT) utilize a zinc finger E-box-binding homeobox 1 (ZEB1)–tissue plasminogen activator (tPA) axis to provide paracrine signals that augment fibroblast recruitment and activation. In reciprocal paracrine signaling, transforming growth factor-β (TGF-β)–activated fibroblasts or IPF fibroblasts (IPFFs) induce RAS activation in ATII cells, which is at least partially driven by secreted protein acidic and rich in cysteine (SPARC). These findings support the concept that aberrant bidirectional epithelial–mesenchymal crosstalk contributes to the development of a profibrogenic microenvironment in which a chronic wound healing response leads to interstitial lung fibrosis.

## Results

### Global transcriptomic changes in fibroblasts exposed to conditioned media from RAS-activated ATII cells identify multiple processes including cell migration

We previously reported that RAS-induced EMT in the human ATII cell line, ATII^ER:KRASV12^ (in which RAS activation can be induced by 4-hydroxytamoxifen [4-OHT]) resulted in production of paracrine factors that augmented TGF-β-induced profibrogenic responses in lung fibroblasts (6). To determine if, and how, fibroblasts responded to epithelial signals in the absence of profibrogenic TGF-β signaling, we characterized the global transcriptomic changes in MRC5 lung fibroblasts exposed to conditioned media (CM) from control or RAS-induced ATII cells by performing RNA-Seq.

**Figure 1:**
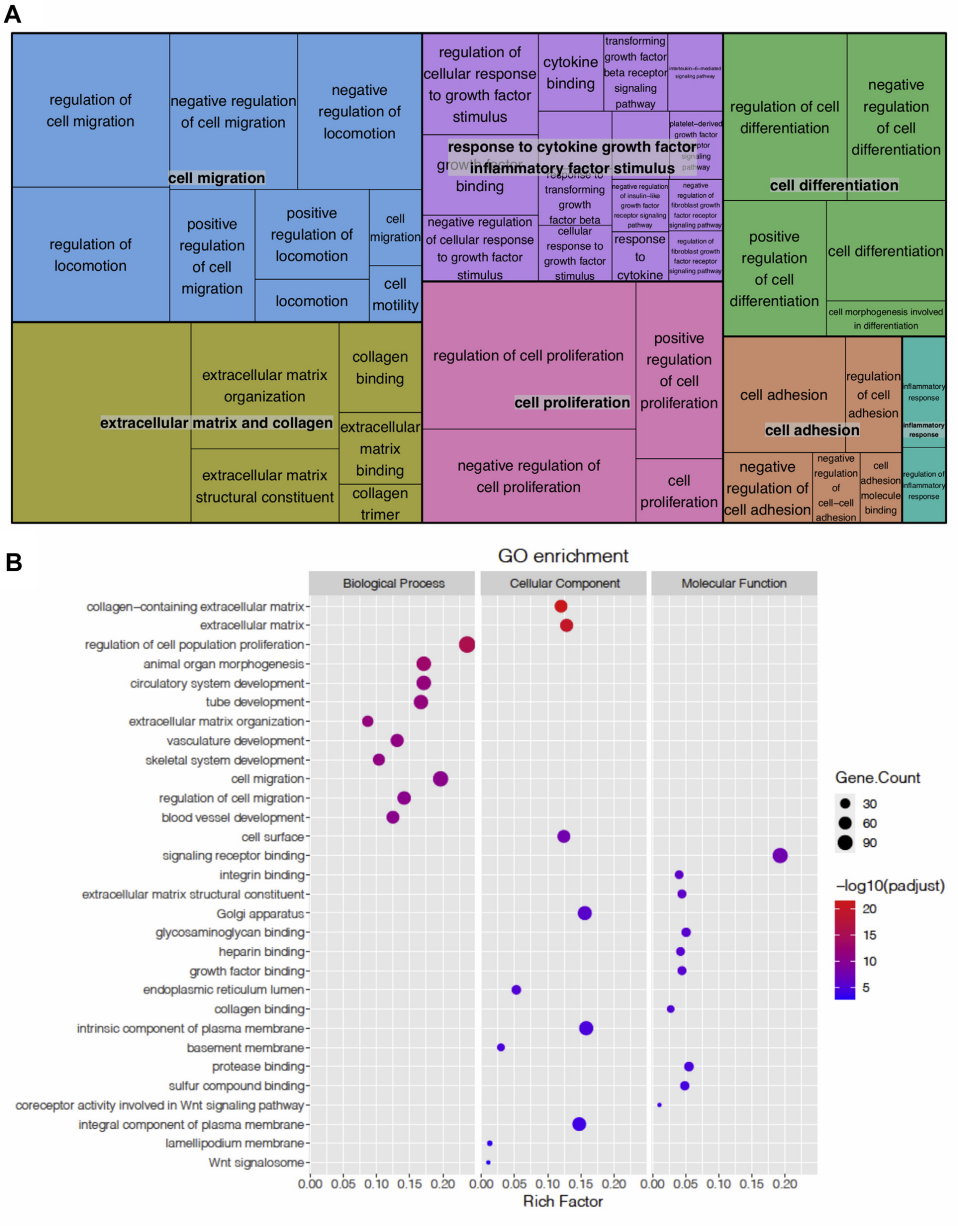
**Global transcriptomic changes in fibroblasts exposed to CM from RAS-activated ATII cells.***A*, REVIGO TreeMap showing Gene Ontology (GO) analysis of upregulated DEGs in MRC5 lung fibroblasts treated with CM from 4-OHT-activated *versus* control ATII^ER:KRASV12^ cells. Common colors represent groupings based on parent GO terms, and each rectangle is proportional to the relative enrichment of the GO term compared with the whole genome. Genes with false discovery rate (FDR) <0.05 were considered as DEGs. *B*, scatter plot showing the top ten enriched GO terms from three categories (biological process, cellular component, and molecular function) according to rich factors. Rich factor is the percentage of DEG-enriched gene count in the given annotated GO terms. The sizes of *circles* represent gene counts, and the colors of *circles* represent the −Log_10_ of the adjusted *p* values. ATII, alveolar epithelial type II; DEGs, differentially expressed genes; CM, conditioned media; 4-OHT, 4-hydroxytamoxifen.

Genes with a false discovery rate (FDR)–adjusted *p* value less than 0.05 were considered as differentially expressed genes (DEGs). In total, 966 DEGs were identified, including 491 upregulated (Table S1) and 475 downregulated (Table S2) genes. We then performed Gene Ontology (GO) enrichment analysis of the identified DEGs using PANTHER (http://pantherdb.org/) (7). The results were grouped into molecular function, biological process, and cellular component. Interestingly, several IPF-related pathological terms were identified, including ECM and collagen, cell migration, and response to cytokine growth factor inflammatory stimulus, which included TGF-β receptor signaling (FDR <0.05; Figs. 1*A* and S1; Tables S3 and S4). Of these, cell migration and collagen containing ECM were among the top ten ranked GO terms from the biological process category (Fig. 1*B*). These results extend our previous report (6) by demonstrating that CM from RAS-activated ATII cells regulates the expression of many genes that augment fibroblast activation and migration independent of exogenous TGF-β. Of note, under these conditions, we did not see changes in expression of *ACTA2*, encoding α-smooth muscle actin (α-SMA) (adjusted *p* value equals 0.289), suggesting that the fibroblasts did not adopt a myofibroblast phenotype under the conditions tested.

**Figure 2:**
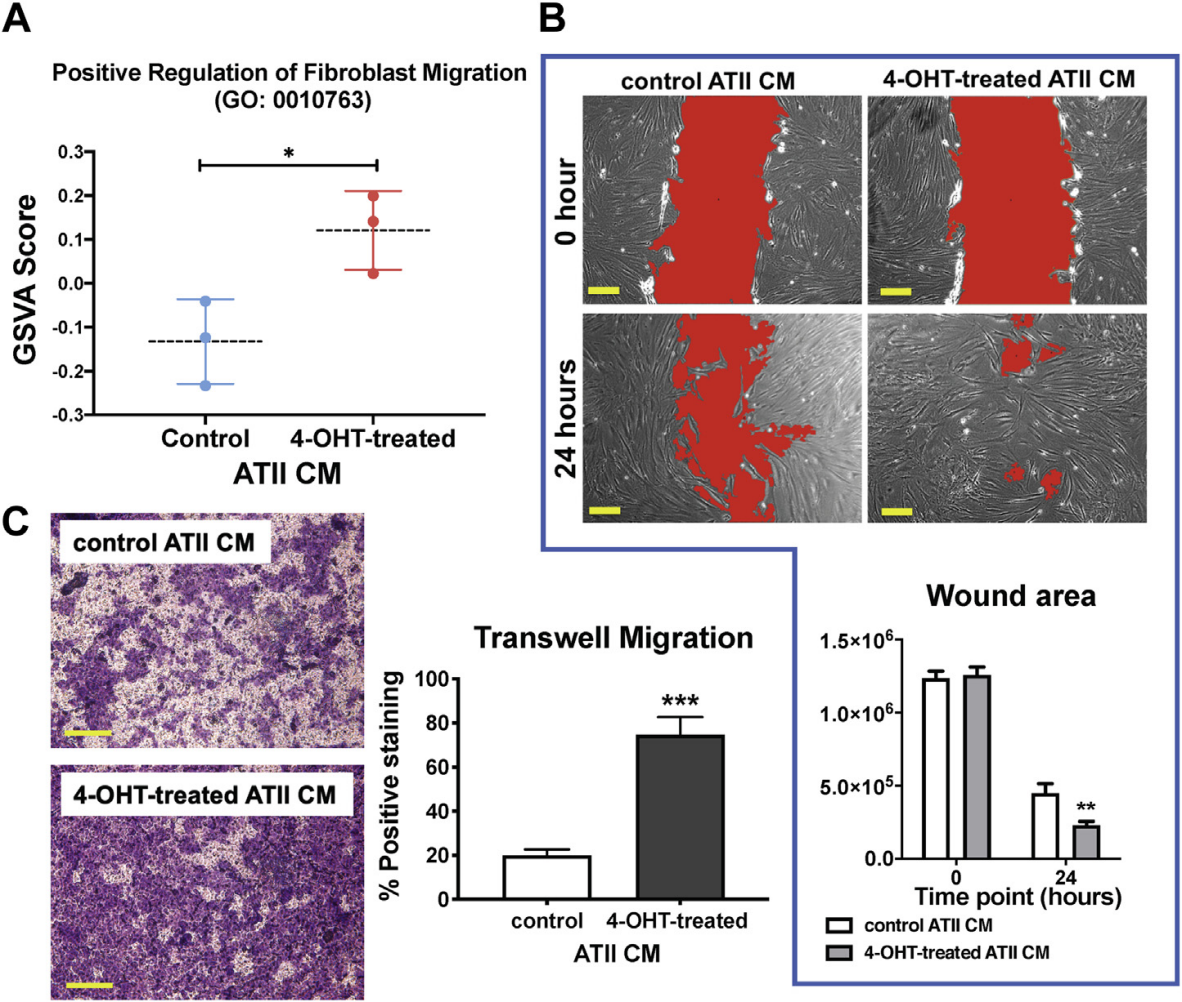
**RAS-activated ATII cells augment fibroblast migration *via* paracrine signaling.***A*, graph showing GSVA scores calculated based on a gene list from GO:0010763 (positive regulation of fibroblast migration) in MRC5 cells treated with CM from control or 4-OHT-activated ATII^ER:KRASV12^ cells. Data are mean  ±  SD; *n* = 3. ∗*p* < 0.05. *B*, scratch wound assay of fibroblasts treated with CM from control or 4-OHT-activated ATII^ER:KRASV12^ cells. Representative images of IPF fibroblasts with the indicated treatments at time 0 or 24 h after the scratch wound. Wounds have been artificially colored *red* to aid visualization. The scale bar represents 200 μm. Graph shows the areas of wounds evaluated with ImageJ, and data are mean  ±  SD; *n*  =  3. ∗∗*p* <  0.01. *C*, transwell migration assays in MRC5 fibroblasts with indicated treatment. Cells were stained with crystal violet. The scale bar represents 100 μm. Data are mean  ±  SD; *n* =  3. ∗∗∗*p*  <  0.001. ATII, alveolar epithelial type II; CM, conditioned media; GSVA, gene set variation analysis; IPF, idiopathic pulmonary fibrosis; 4-OHT, 4-hydroxytamoxifen.

The effect of CM from RAS-induced ATII cells on genes controlling cell migration was further demonstrated using gene set variation analysis (GSVA) with a gene list from GO:0010763 (positive regulation of fibroblast migration); the GSVA score calculated based on this gene list was higher in MRC5 cells incubated with CM from 4-OHT-treated *versus* control ATII^ER:KRASV12^ cells (*p* < 0.05; Fig. 2*A*). To validate this finding on cell migration, scratch wound assays were performed using MRC5 or IPFFs treated with CM from control or 4-OHT-activated ATII^ER:KRASV12^ cells. Twenty-four hours after the scratch wound, IPFFs treated with CM from 4-OHT-activated ATII cells almost filled all the gaps, whereas the wound in the control cells was still open (*p* < 0.01; Fig. 2*B*). Similar results were obtained using MRC5 lung fibroblasts (Fig. S2*A*). Under the same conditions, CM from RAS-activated ATII cells did not affect the cell viability in MRC5 (Fig. S2*B*). In addition, in a transwell migration assay, a 3.5-fold increase in migration was detected using MRC5 lung fibroblasts treated with CM from 4-OHT-activated ATII^ER:KRASV12^ cells compared with those treated with CM from control cells over a 24-h period (*p* < 0.001; Fig. 2*C*). Together, these results demonstrate that RAS-activated ATII cells augment fibroblast migration *via* paracrine signaling.

### RAS-activated ATII cells augment fibroblast migration *via* paracrine signaling, in which ZEB1 and tPA are key regulators

**Figure 3:**
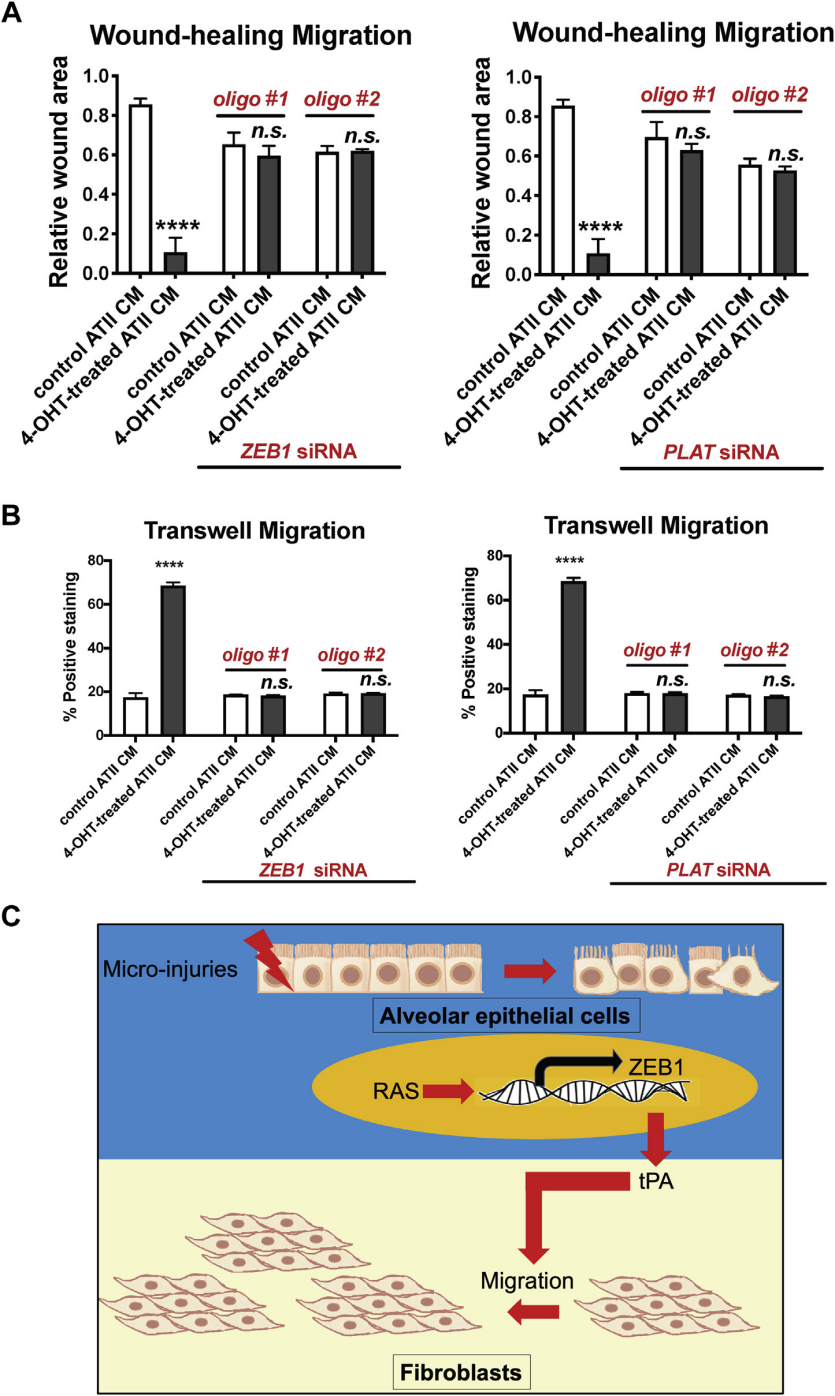
**RAS-activated ATII cells augment fibroblast migration *via* paracrine signaling, in which ZEB1 and tissue plasminogen activator (tPA, encoded by gene *PLAT*) are key paracrine regulators.***A*, scratch wound assay of fibroblasts treated with CM from ATII^ER:KRASV12^ cells with indicated treatment. Graph showing the areas of wounds evaluated with ImageJ, and data are mean ± SD; *n*  =  3. ∗∗∗∗*p* < 0.0001. n.s. (not significant) *p* > 0.05. *B*, transwell migration assays in fibroblasts treated with CM from ATII^ER:KRASV12^ cells with indicated treatment. Graph showing the percentage of positive staining of fibroblasts with indicated treatment evaluated with ImageJ, and data are mean ± SD; *n*  =  3. ∗∗∗∗*p* < 0.0001. n.s. *p* > 0.05. *C*, diagram summarizing a key role of tPA secreted by RAS-activated ATII cells in augmenting fibroblast migration *via* paracrine signaling. ATII, alveolar epithelial type II; CM, conditioned media; ZEB1, zinc finger E-box-binding homeobox 1.

As we previously demonstrated that ZEB1 and its transcriptional target, tPA (encoded by *PLAT* gene) are key regulators of epithelial–mesenchymal crosstalk (6), we hypothesized that these two proteins may also control the ability of RAS-activated ATII cells to promote migration and recruit fibroblasts by paracrine signaling. First, we depleted *ZEB1* in ATII^ER:KRASV12^ cells *via* RNAi (Fig. S3*A*). We found that this completely abolished the promigratory effects of CM from RAS-activated ATII cells on lung fibroblasts in both wound healing assay (*p* > 0.05; Figs. 3*A* and S3*B*) and transwell migration assay (*p* > 0.05; Fig. 3*B*). Next, we determined whether tPA was a key paracrine regulator of fibroblast migration. Recombinant tPA treatment significantly enhanced fibroblast migration (*p* < 0.0001; Fig. S3*C*). Similar to *ZEB1*, CM was collected from control or *PLAT*-depleted ATII^ER:KRASV12^ cells treated without or with 4-OHT (Fig. S3, *D* and *E*). As expected, RAS-activated ATII cells augmented fibroblast migration in both wound healing assay (*p* < 0.0001; Figs. 3*A* and S3*F*) and transwell migration assay (*p* < 0.0001; Fig. 3*B*), whereas under the same conditions, depletion of *PLAT* in ATII^ER:KRASV12^ cells completely abolished the promigratory effects of CM from RAS-activated ATII cells on lung fibroblasts (*p* > 0.05; Figs. 3, *A* and *B* and S3*F*). Taken together, these data highlight the importance of ZEB1 and tPA as key regulators of epithelial–mesenchymal crosstalk to promote fibroblast recruitment, as well as fibroblast activation, as demonstrated previously (6) (Fig. 3*C*).

### TGF-β-activated fibroblasts or IPFFs induce RAS activation in ATII cells

**Figure 4:**
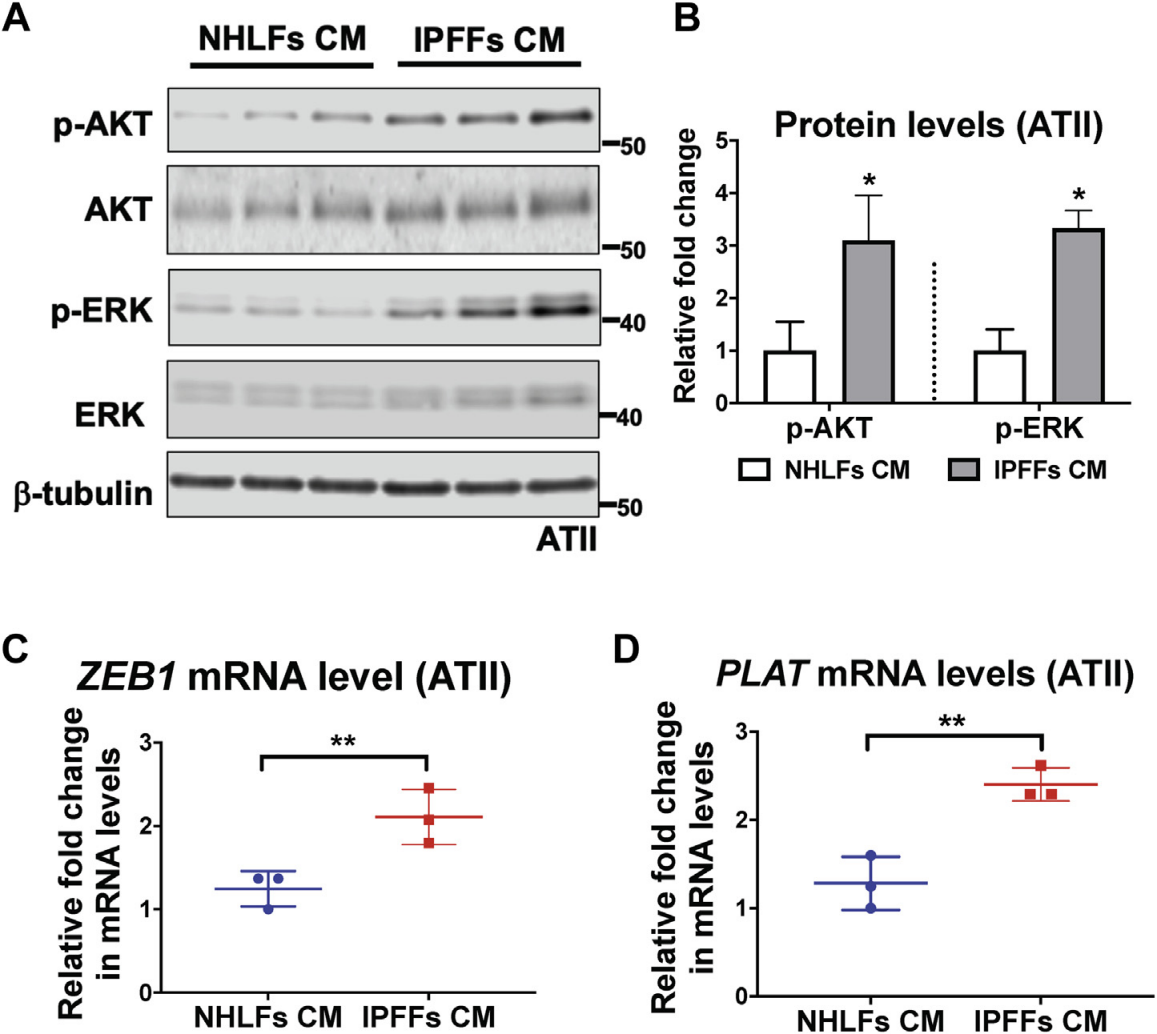
**IPF fibroblasts induce RAS activation in ATII cells *via* paracrine signaling.***A*, protein expression of phospho-AKT (p-AKT), AKT, phospho-ERK (p-ERK), and ERK in ATII cells treated with indicated CM from NHLFs or IPFFs. β-tubulin was used as a loading control. *B*, graph showing protein levels of p-AKT or p-ERK in ATII cells treated with CM from NHLFs or IPFFs. Total AKT or ERK-normalized protein levels in ATII cells treated with CM from NHLFs were used to set the baseline value at unity. Data are mean ± SD; *n* = 3. ∗*p* < 0.05. *C* and *D*, fold change in mRNA levels of *ZEB1* (*C*) or *PLAT* (*D*) in ATII cells treated with CM from NHLFs or IPFFs. β-actin–normalized mRNA levels in ATII cells treated with CM from NHLFs were used to set the baseline value at unity. Data are mean ± SD; *n* = 3 samples per group. ∗∗*p* < 0.01. ATII, alveolar epithelial type II; CM, conditioned media; ERK, extracellular-regulated kinase; IPF, idiopathic pulmonary fibrosis; IPFFs, IPF fibroblasts; NHLFs, normal human lung fibroblasts.

Our current and previous (6) findings suggest that a ZEB1–tPA axis is involved in the paracrine signaling between RAS-activated ATII cells and fibroblasts to augment not only their recruitment but also their activation in the presence of TGF-β. To explore whether this epithelial–mesenchymal crosstalk was bidirectional, we investigated the effect of the activated fibroblasts on the alveolar epithelium. First, MRC5 lung fibroblasts were left untreated or treated with TGF-β (Fig. S4*A*) to induce myofibroblast differentiation, as measured using the myofibroblast marker, α-SMA. An overall increase in α-SMA protein expression manifested after 24 h of TGF-β treatment, with maximal induction being observed by Western blot at 48 h post-TGF-β treatment (Fig. S4*B*). To eliminate the direct effect of exogenous TGF-β in CM collected from fibroblasts on ATII cells, fibroblast cultures were placed in fresh medium without TGF-β for another 24 h before CM was harvested (Fig. S4*A*; group “48 h+, 24 h−”). As shown in Figure S4*B*, fibroblast differentiation into myofibroblasts was maintained under these conditions, evidenced by a comparable level of α-SMA protein level. We then treated ATII cells with CM from control or TGF-β-activated MRC5 cells (Fig. S4*C*). Western blot analysis suggested that RAS signaling was activated upon treatment with CM from TGF-β-activated fibroblasts, reflected by significant increases in phosphorylation of AKT and extracellular-regulated kinase (ERK) (Fig. S4*D*). Of note, TGF-β-activated fibroblast CM also increased ZEB1 expression in ATII cells (Fig. S4*D*). Since it has been reported that IPFFs have a higher level of autocrine TGF-β activation (8), we next compared the effects of CM from normal human lung fibroblasts (NHLFs) or IPFFs on ATII cells. We found that RAS–ZEB1 signaling was significantly higher upon treatment with CM from IPFFs, reflected by significantly higher increases in phosphorylation of AKT and ERK levels (*p* < 0.05; Fig. 4, *A* and *B*) as well as *ZEB1* (*p* < 0.01; Fig. 4*C*) and *PLAT* (tPA) expressions (*p* < 0.01; Fig. 4*D*). Together, these results suggest that signaling occurs between fibroblasts and ATII cells leading to RAS activation and induction of ZEB1, and that this effect is augmented by CM from fibroblasts that have been differentiated into myofibroblasts by TGF-β or by CM from IPFFs independently of exogenous TGF-β.

### SPARC, a TGF-β-induced secreted protein, is highly expressed in IPF

**Figure 5:**
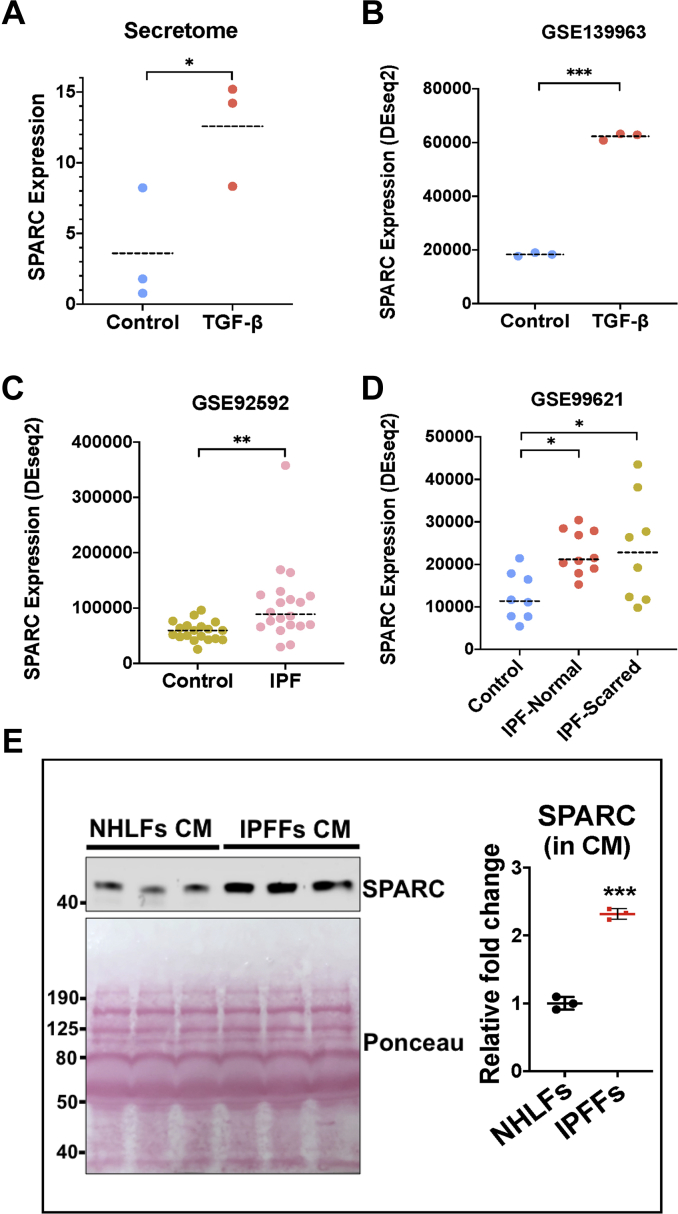
**SPARC, a TGF-β-induced secreted protein, is highly expressed in IPF.***A*, quantitative secretome analysis identifies an increased level of SPARC in the CM from TGF-β-treated MRC5 cells. Data are mean ± SD; *n* = 3. ∗*p* < 0.05. *B*, SPARC expression in human lung fibroblasts treated with or without TGF-β (GSE139963). ∗∗∗*p* < 0.001. *C*, SPARC expression in lung tissues from healthy controls and IPF patients (GSE92592). ∗∗*p* < 0.01. *D*, SPARC expression in lung tissues from healthy controls and IPF samples with macroscopically normal-appearing (IPF-normal) or scarred (IPF-scarred) (GSE99621). ∗*p* < 0.05. *E*, protein expression of SPARC in CM from NHLFs or IPFFs. Ponceau S staining showing total protein levels. Graph showing SPARC protein levels in CM from NHLFs or IPFFs. Data are mean ± SD; *n* = 3. ∗∗∗*p* < 0.001. CM, conditioned media; IPF, idiopathic pulmonary fibrosis; IPFFs, IPF fibroblasts; NHLFs, normal human lung fibroblast; SPARC, secreted protein acidic and rich in cysteine; TGF-β, transforming growth factor-β.

As CM derived from TGF-β-activated fibroblasts or IPFFs induced RAS signaling in ATII cells, we next investigated which secreted factors from activated fibroblasts were responsible for this effect. By performing quantitative proteomic analysis of the CM from control or TGF-β-treated MRC5 cells, we identified 15 secreted proteins whose levels were upregulated upon TGF-β treatment (Table 1; Tables S5 and S6). Of these, SPARC was among the top candidates (Fig. 5*A*; Table 1), suggesting a potential regulatory role of SPARC in mesenchymal–epithelial crosstalk driven by TGF-β. This was further validated in a publicly available dataset (GSE139963) (9), in which RNA-Seq was performed in human lung fibroblasts treated with or without TGF-β. Potential TGF-β-induced secreted proteins were identified by overlapping the upregulated genes with a list of secreted proteins (predicted) from Human Protein Atlas (https://www.proteinatlas.org/humanproteome/tissue/secretome). We identified 116 predicted secreted proteins that were significantly elevated (FDR-adjusted *p* value <0.05 and Log_2_[fold change] (Log_2_[fold change])  >1) in TGF-β-treated lung fibroblasts compared with controls (Fig. S5; Table S7), and the expression of SPARC was increased by over 3-fold (*p* < 0.001) (Fig. 5*B*; Table 1; Fig. S5*B*; Table S7). The importance of SPARC in IPF pathogenesis was further confirmed in other datasets. In dataset GSE92592 (10), SPARC levels are increased in IPF tissues compared with control lungs (*p* < 0.01; Fig. 5*C*; Table 1), consistent with our recent report (11). Further analysis of GSE99621 (12) showed that SPARC expression is increased not only in scarred but also in those macroscopically unaffected (normal-appearing) regions of IPF lung (*p* < 0.05; Fig. 5*D*; Table 1). In addition, we confirmed a significantly enhanced secretion of SPARC in CM from IPFFs *versus* NHLFs by Western blotting (*p* < 0.001; Fig. 5*E*). Therefore, these data suggested that SPARC is induced as an early response to fibroblast activation and that it may be associated with IPF disease progression.

**Table 1:** List of secreted proteins in the CM that are upregulated upon TGF-β treatment in MRC5 cells identified by quantitative proteomic analysis and their expressions in GSE139963, GSE92592, and GSE99621

Protein	Secretome		GSE139963		GSE92592		GSE99621			
	TGF-β *versus* control		TGF-β *versus* control		IPF *versus* control		IPF—normal *versus* control		IPF—scarred *versus* control	
	*p*	Log_2_FC	FDR	Log_2_FC	FDR	Log_2_FC	FDR	Log_2_FC	FDR	Log_2_FC
A2M	**1.08E − 02**	5.44	**0.00E + 00**	−3.74	**1.76E − 08**	1.01	**2.45E − 03**	0.80	2.16E − 01	0.41
IGFBP3	**3.94E − 04**	4.70	**1.22E − 89**	6.14	**1.21E − 04**	1.01	6.50E − 01	0.20	7.33E − 01	0.17
SERPINE2	**1.84E − 03**	4.11	**0.00E + 00**	3.85	**1.26E − 15**	2.37	**6.26E − 05**	1.53	**2.59E − 04**	1.49
PXDN	**1.29E − 04**	3.51	**5.92E − 235**	1.63	**8.00E − 05**	0.77	1.24E − 01	0.40	**1.26E − 03**	0.77
PGS1	**1.29E − 03**	2.06	**8.44E − 04**	−0.32	4.85E − 01	−0.08	7.74E − 02	−0.42	**2.16E − 02**	−0.55
THBS1	**2.69E − 02**	1.98	**0.00E + 00**	2.02	**4.37E − 02**	1.01	8.57E − 01	−0.12	8.95E − 01	−0.10
SPARC	**4.75E − 02**	1.80	**0.00E + 00**	1.76	**1.37E − 04**	0.87	**4.58E − 03**	0.89	**2.86E − 04**	1.15
APOA1-AS	**2.43E − 02**	1.79	2.02E − 01	−0.37	**9.27E − 08**	−1.54	**5.36E − 07**	−2.51	**1.70E − 05**	−2.31
COL4A2	**2.06E − 02**	1.47	**0.00E + 00**	2.98	**1.25E − 04**	0.91	8.00E − 01	0.14	2.54E − 01	0.49
TGFBI	**1.20E − 03**	1.41	**1.17E − 221**	1.76	**5.91E − 07**	0.90	**4.28E − 03**	0.90	**2.50E − 03**	0.99
MMP2	**3.99E − 02**	1.11	**9.86E − 108**	1.16	**9.10E − 18**	2.04	**1.84E − 13**	1.32	**3.05E − 13**	1.38
FN1	**1.14E − 03**	1.00	**0.00E + 00**	1.76	5.86E − 02	0.52	**2.56E − 07**	1.04	**3.33E − 09**	1.23
IGFBP7	**9.63E − 04**	0.66	**3.29E − 99**	1.74	**7.19E − 09**	0.92	**6.31E − 16**	1.42	**8.76E − 20**	1.66
COL12A1	**4.19E − 02**	0.51	6.97E − 01	−0.04	**1.57E − 02**	0.51	2.63E − 01	0.37	3.94E − 01	0.32
CYCS	**1.77E − 03**	0.40	**6.29E − 10**	0.52	2.62E − 01	0.15	8.97E − 01	0.05	8.84E − 01	0.06

Abbreviation: FC, fold change.

Numbers in bold mean *p* values less than 0.05 and are statistically significant.

### SPARC is a key fibroblast-derived paracrine regulator of RAS activation in ATII cells

**Figure 6:**
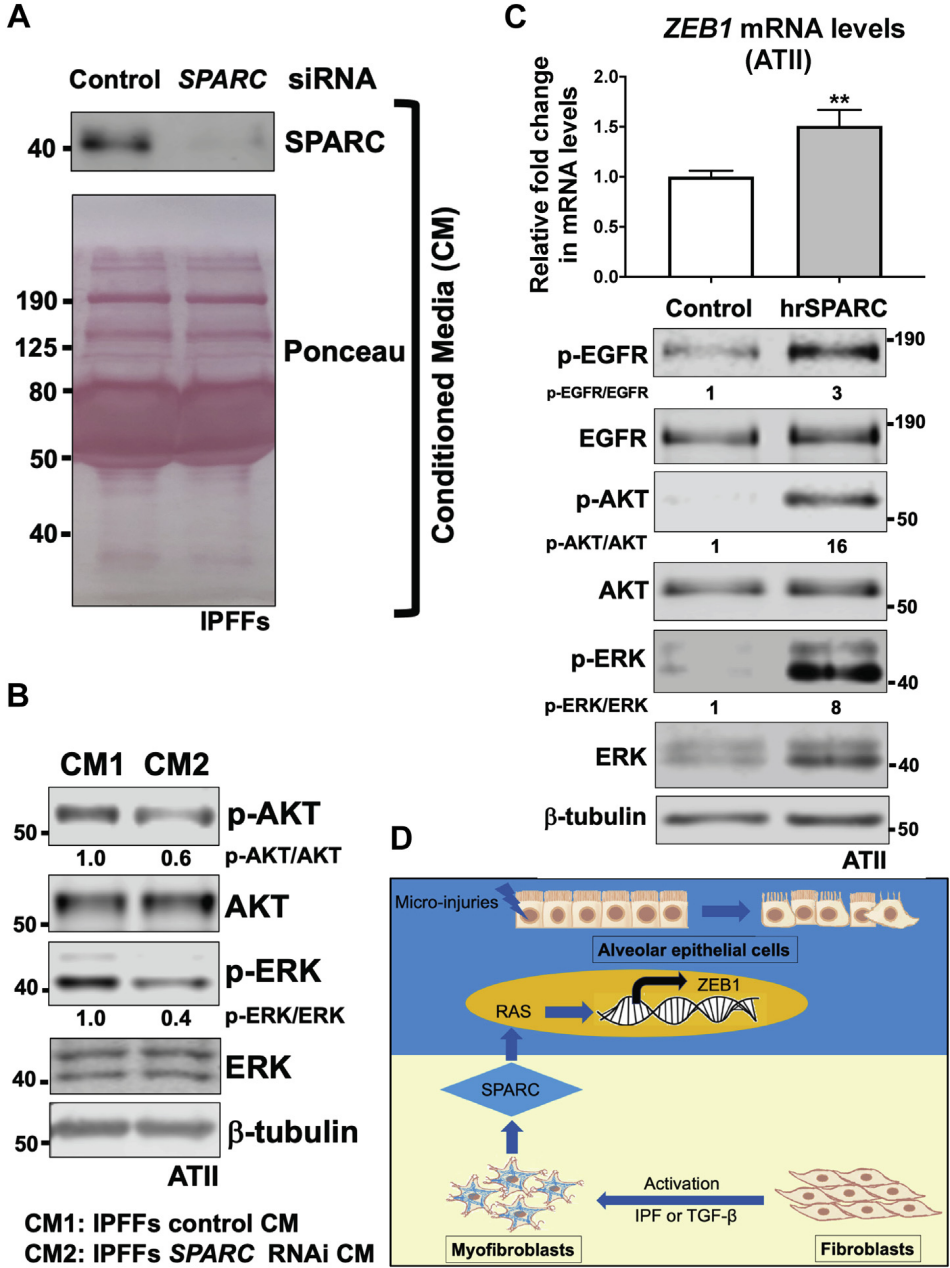
**SPARC is a key fibroblast-derived paracrine regulator of RAS activation in ATII cells.***A*, protein expression of SPARC in CM from IPFFs with indicated treatment. Ponceau S staining showing total protein levels. *B*, protein expression of phospho-AKT (p-AKT), AKT, phospho-ERK (p-ERK), and ERK in ATII cells treated with CM from IPFFs with indicated treatment. β-tubulin was used as a loading control. Total AKT or ERK-normalized protein levels in ATII cells treated with CM from control IPFFs were used to set the baseline value at unity. *C*, protein expression of phospho-EGFR (p-EGFR), EGFR, p-AKT, AKT, p-ERK, and ERK in ATII cells treated with or without human recombinant SPARC (hrSPARC). β-tubulin was used as a loading control. Total EGFR, AKT, or ERK-normalized protein levels in control cells were used to set the baseline value at unity. Fold change in mRNA levels of *ZEB1* in ATII cells treated with or without hrSPARC. β-actin–normalized mRNA levels in control cells were used to set the baseline value at unity. Data are mean ± SD; *n* = 3 samples per group. ∗∗*p* < 0.01. *D*, diagram summarizing SPARC as a key fibroblast-derived paracrine regulator of RAS activation in ATII cells. ATII, alveolar epithelial type II; CM, conditioned media; EGFR, epithelial growth factor receptor; ERK, extracellular-regulated kinase; IPFFs, IPF fibroblasts; SPARC, secreted protein acidic and rich in cysteine; *ZEB1*, zinc finger E-box-binding homeobox 1.

We recently reported that paracrine SPARC signaling from IPFFs dysregulates alveolar epithelial barrier integrity (11). We therefore hypothesized that SPARC might also mediate RAS activation in ATII cells during fibroblast–epithelial crosstalk. To test this hypothesis, ATII cells were exposed to CM harvested from control or *SPARC*-depleted NHLFs treated without or with TGF-β (Fig. S6*A*). As expected, we detected enhanced expression (Fig. S6*B*) and secretion (Fig. S6*C*) of SPARC from TGF-β-activated NHLFs compared with controls, and this was abolished using RNAi. When the CM was applied to ATII cells, CM from TGF-β-activated fibroblasts induced RAS activation, as evidenced by increases of phosphorylation in both AKT and ERK, whereas depletion of *SPARC* completely abolished the effects of CM from TGF-β-activated fibroblasts on ATII cells (Fig. S6*C*). We then compared CM from IPFFs: consistent with our previous results, an increased SPARC secretion was detected in CM from IPFFs *versus* NHLFs (Fig. S6*D*). Furthermore, while RAS signaling in ATII cells was activated by treatment with CM from IPFFs, these effects were completely abolished upon *SPARC* depletion in IPFFs (Fig. 6*A*; Fig. S6*D*), as evidenced by decreased phosphorylation of AKT and ERK levels (Fig. 6*B*; Fig. S6*D*). Furthermore, human recombinant SPARC treatment alone was sufficient to induce epithelial growth factor receptor (EGFR) activation in ATII cells, demonstrated by increased phosphorylation levels of EGFR and its downstream AKT and ERK phosphorylation levels (Fig. 6*C*). EGFR activation was accompanied by a significant increase in mRNA expression of *ZEB1* (Fig. 6*C*) and a reduction in *CDH1* (E-cadherin) (Fig. S6*E*). Therefore, our results strongly suggest that SPARC is a critical paracrine regulator from activated lung fibroblasts that induces RAS activation in ATII cells (Fig. 6*D*).

## Discussion

**Figure 7:**
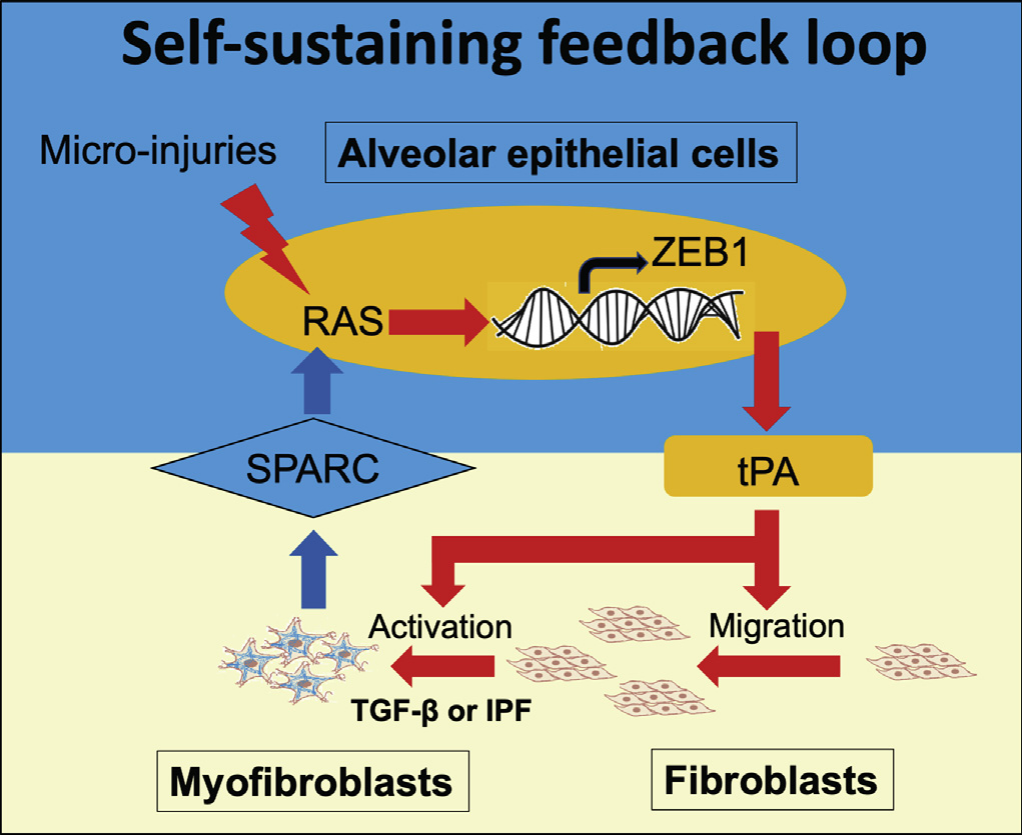
**Diagram summarizing a critical role of bidirectional epithelial–mesenchymal crosstalk in pulmonary fibrosis (details provided in**Discussion**section)**.

IPF is a chronic progressive lung disease with limited therapeutic options (1, 13). Abnormal wound healing responses appear to make major contributions to the scarring process, but the underlying pathological mechanisms are unclear. Similar to studies in kidney fibrosis (14), our findings support the concept that aberrant epithelial–mesenchymal crosstalk contributes to the development of interstitial lung fibrosis. Our studies are consistent with the establishment of a bidirectional profibrogenic positive feedback loop, which maintains a chronic wound environment involving activated epithelial cells and fibroblasts that drive fibrosis progression rather than wound resolution (Fig. 7).

Epithelial cells undergo EMT in response to injury, allowing them to adopt a repair phenotype, which involves signaling to other cell types including inflammatory cells and resident mesenchymal cells. We previously reported that ATII cells undergoing RAS-induced EMT secrete tPA to augment TGF-β-induced myofibroblast differentiation and that ZEB1 is a key regulator of this paracrine signaling (6). To investigate further whether fibroblasts can respond to epithelial-derived signals in the absence of TGF-β, we used RNA-Seq to study the global transcriptomic changes in lung fibroblasts exposed to CM from ATII cells undergoing RAS-induced EMT. Using this unbiased approach, several potential mechanisms of relevance to IPF pathology were identified, including ECM and collagen, cell migration, cytokine binding, and inflammatory response, which included changes in expression of genes associated with TGF-β receptor signaling pathways. While we observed upregulation of ECM and collagen genes including *COL3A1*, *COL4A6*, *COL14A1*, and *COL18A1* in the transcriptomic dataset, we did not observe changes in expression of either *COL1A1*, the major interstitial collagen produced in response to TGF-β stimulation, or *ACTA2*, which encodes alpha-smooth muscle actin, a marker of myofibroblast differentiation (Table S1; GSE163908). This suggests that the magnitude of TGF-β receptor signaling caused by the epithelial-derived CM was insufficient to drive differentiation of the fibroblasts into myofibroblasts. These findings are consistent with our previous studies (6) where exogenous TGF-β was required to drive myofibroblast differentiation. However, our observation of upregulation of genes associated with TGF-β receptor signaling and increased expression of many profibrotic genes in fibroblasts following exposure to CM from epithelial cells undergoing RAS-induced EMT may help explain why the CM augmented the effects of exogenous TGF-β that we observed previously (6).

Building on the strong gene expression signature for cell migration, we confirmed induction of a migratory phenotype in fibroblasts exposed to epithelial CM and demonstrated that this involved a ZEB1–tPA signaling axis, similar to that required for augmentation of TGF-β-induced myofibroblast differentiation (6). Fibroblasts from pulmonary fibrosis have altered pathological properties (15) including enhanced migration compared with fibroblasts from control lungs (16, 17). In our studies, we found that CM produced by ATII cells undergoing RAS-induced EMT markedly induced fibroblast migration and recruitment. The importance of the ZEB1–tPA axis in this process was then confirmed, with their silencing abolishing the promigratory effects of CM from RAS-activated ATII cells on lung fibroblasts. We previously identified that ZEB1 is expressed in epithelial cells of thickened alveolar septae where ECM deposition is evident (6). This suggests that ZEB1 is induced as an early response to alveolar epithelial injury and that, by regulating tPA expression, ZEB1 may promote fibroblast recruitment and thereby increase their numbers in the wound site. Consistent with our findings, it has been reported that neutralizing antibodies against tPA inhibit fibroblast migration (18). Mechanistically, tPA has direct cellular effects by virtue of its ability to bind to the low-density lipoprotein receptor–related protein-1, triggering low-density lipoprotein receptor–related protein-1 tyrosine phosphorylation and recruitment of β1-integrin signaling involving integrin-linked kinase (19, 20).

While the activation of the ZEB–tPA axis may be a normal physiological response to injury, deregulation of this axis may sensitize the underlying fibroblasts to drive a pathological profibrogenic response (21). Consistent with this concept, in kidney studies, microinjuries on renal epithelium have been proposed to create a profibrotic microenvironment *via* TGF-β or connective tissue growth factor (14, 22), promoting the aggregation of myofibroblasts, and in return, myofibroblasts may enhance the apoptosis of epithelial cells by secreting reactive oxygen species (23, 24) or angiotensin II (25, 26), so generating self-sustained pathological feedbacks. We used CM harvested from TGF-β-activated fibroblasts or IPFFs and found that both enhanced RAS activation and induction of ZEB1 in ATII cells *via* paracrine signaling. Moreover, we found that CM from IPFFs induced significantly more RAS activation and ZEB1 expression than normal fibroblasts in the absence of exogenous TGF-β, pointing to a pathological mechanism within the IPFF population. This finding may be explained by a previous report that autocrine TGF-β signaling is enhanced in IPFFs (8). Taken together, these data suggest that bidirectional epithelial–mesenchymal crosstalk may become dysregulated in IPF leading to fibrosis progression rather than wound resolution.

Using proteomics and bioinformatics, we were able to identify SPARC, secreted by TGF-β-activated fibroblasts, as a key paracrine regulator of RAS activation in ATII cells. SPARC, a cysteine-rich acidic matrix-associated protein, containing three epithelial growth factor–like repeats, is required for the collagen in bone to become calcified but is also involved in ECM synthesis and promotion of changes to cell shape (27, 28, 29, 30, 31, 32, 33, 34). Consistent with our recent findings (11), our analysis based on publicly available transcriptome datasets indicate that SPARC expression is increased not only in scarred regions but also in macroscopically unaffected (normal-appearing) regions of IPF lung tissue (10, 12). This suggests that SPARC is induced as an early response to fibroblast activation. We found that while SPARC expression can be induced by TGF-β in normal lung fibroblasts, it is aberrantly expressed by IPFFs and that it acts *via* a paracrine mechanism to mediate RAS activation in ATII cells. SPARC has been shown by several studies to be able to regulate ERK signaling (35, 36, 37), although the upstream mechanism remains to be elucidated. In this study, our results demonstrated that SPARC alone is sufficient to induce EGFR activation in ATII cells, suggesting that SPARC might signal *via* the EGFR similar to tenascin C (38, 39, 40) *via* their epithelial growth factor–like repeats (41). Together, we have provided strong evidence suggesting that in both TGF-β-activated healthy lung fibroblasts and IPFFs, paracrine SPARC signaling dysregulates alveolar epithelial barrier integrity (11) and activates EGFR/RAS signaling in ATII cells to maintain a chronic wound-healing phenotype.

In summary, we identify a mechanism whereby bidirectional epithelial–mesenchymal crosstalk results in self-sustaining activation of wound healing responses. This involves a ZEB1–tPA axis in ATII cells, which contributes to paracrine signaling that supports recruitment of fibroblasts and augments TGF-β-induced fibrotic responses; in turn, the aberrant behavior of IPFFs (or normal fibroblasts activated by high levels of TGF-β) promotes persistent alveolar epithelial activation *via* paracrine mediators including SPARC, which prevents resolution of normal epithelial repair responses and restoration of tissue homeostasis. Such a chronic wound environment may drive disease progression in pulmonary fibrosis (Fig. 7).

## Experimental procedures

### Lung tissue sampling

All human lung tissue samples for primary cell culture were approved by the Southampton and South West Hampshire and the Mid and South Buckinghamshire Local Research Ethics Committees, and all subjects gave written informed consent. The study abided by the Declaration of Helsinki principles. Clinically indicated IPF lung biopsy tissue samples and nonfibrotic control tissue samples (macroscopically normal lung sampled remote from a cancer site) were assessed as surplus to clinical diagnostic requirements. All IPF samples were from patients subsequently receiving a multidisciplinary diagnosis of IPF according to international consensus guidelines (42).

### Cell culture, reagents, and transfections

Primary parenchymal lung fibroblast cultures were established from IPF or control lung tissues as described previously (6, 43, 44, 45). MRC5 lung fibroblasts were obtained from the European Collection of Authenticated Cell Cultures. Fibroblasts were cultured in Dulbecco's modified Eagle's medium supplemented with 10% fetal bovine serum, 50 units/ml penicillin, 50 μg/ml streptomycin, 2 mM l-glutamine, 1 mM sodium pyruvate, and 1× nonessential amino acids (all from Life Technologies). ATII^ER:KRASV12^ cells (6, 45, 46, 47) were cultured in DCCM-1 (Biological Industries, Ltd) supplemented with 10% newborn calf serum (Life Technologies), 1% penicillin, 1% streptomycin, and 1% l-glutamine (all from Life Technologies). All cells were kept at 37 °C and 5% CO_2_. No *mycoplasma* contamination was detected in the cell lines used.

siRNA oligos against *ZEB1* (MU-006564-02-0002, Set of four Upgrade) and *PLAT* (tPA) (MU-005999-01-0002, Set of four Upgrade) and *SPARC* (L-003710-00-0005; M-003710-02-0005) were purchased from Dharmacon. Sequences are available from Dharmacon or on request. As a negative control, we used siGENOME RISC-Free siRNA (Dharmacon; D-001220–01). ATII^ER:KRASV12^ cells were transfected with the indicated siRNA oligos at a final concentration of 35 nM using DharmaFECT 2 reagent (Dharmacon). Fibroblasts were transfected with the indicated siRNA oligos at the same concentration using Lipofectamine RNAiMAX reagent (Invitrogen). Recombinant tPA protein was from Bio-Techne, and human recombinant SPARC was from Peprotech.

### RNA isolation, library construction, and sequencing

To identify global transcriptomic changes in fibroblasts exposed to CM from RAS-activated ATII cells, RNA-Seq was performed in MRC5 cells treated with either CM from control or 4-OHT-activated ATII^ER:KRASV12^ cells. In brief, total RNA was isolated using RNeasy mini kit (Qiagen) according to the manufacturer's instructions and quantified using a Nanodrop Spectophotometer 2000c (Thermo Fisher Scientific). A total amount of 3 μg RNA per sample was used as input material for library construction. Sequencing libraries were generated using NEBNext UltraTM RNA Library Prep Kit for Illumina (NEB) following the manufacturer's instruction. Libraries were pooled in equimolar and sequenced using the paired-end strategy (2 × 150) on the Illumina NovaSeq 6000 platform following the standard protocols (Novogene).

### RNA-Seq data analysis

Three publicly available datasets (GSE139963 (9), GSE92592 (10), and GSE99621 (12)) were collected from Gene Expression Omnibus. GSE139963 was in GPL18573 platform, Illumina NextSeq 500 (*Homo sapiens*), which composed of human lung fibroblasts treated with PBS (control) or TGF-β in triplicates. GSE92592 was in GPL11154 platform, Illumina HiSeq 2000 (*H. sapiens*), which consisted of 20 IPF and 19 control lung samples. GSE99621 was in GPL16791 platform, Illumina HiSeq 2500 (*H. sapiens*), which included 26 samples from indicated sections (eight from healthy lungs, ten from IPF-unaffected lung tissues, and eight from IPF scared areas). Quality control of GSE163908, GSE139963, GSE92592, and GSE99621 data was performed using FastQC (http://www.bioinformatics.babraham.ac.uk/projects/fastqc) and MultiQC (48). Trim Galore (https://github.com/FelixKrueger/TrimGalore) was used to trim adapters, reads with low quality (<30), and short length (<50 bp). RNA-Seq reads were mapped to Human genome Ensembl GRCh38 using Hisat2 (49) (version 2.1.0) with default codes. Sam files were transformed into bam files using samtools (50) (version 1.9). The read counts of each gene were summarized using featureCounts (51) (version 1.6.5). Raw read counts were imported into RStudio (version 3.6.1) and analyzed by using R package of DESeq2 (52) (version 1.26.0). Transcripts with low abundance (under ten counts across all samples) were removed. Genes with FDR *p* value less than 0.05 adjusted by using Benjamini–Hochberg method were considered as DEGs.

### Pathway enrichment analysis

GO enrichment analysis was generated through PANTHER (7) Web site. REVIGO TreeMap was generated *via* an online tool available at http://revigo.irb.hr/ (53). Parameters were set as FDR <0.05. GSVA (54) was used to calculate pathway activity.

### Western blot analysis

Western blot analysis was performed with lysates from cells with urea buffer (8 M urea, 1 M thiourea, 0.5% CHAPS, 50 mM DTT, and 24 mM spermine). Primary antibodies were from Cell Signalling Technology (α-SMA; 14968; Phospho-Smad2, 3104; β-tubulin, 86298; phospho-EGFR, 2234; phospho-AKT, 9271; phospho-ERK, 9101; AKT, 9272; ERK, 9102; and EGFR, 4267), Santa Cruz (ZEB1, sc-25388; SPARC, sc-398419), and Millipore (tPA, 05-883). Signals were detected using an ECL detection system (GE Healthcare) or Odyssey imaging system (LI-COR) and evaluated by ImageJ 1.42q software (National Institutes of Health).

### Quantitative RT-PCR

Real-time quantitative RT-PCR was performed using gene-specific primers (QuantiTect Primer Assays; Qiagen) for *ZEB1* (QT00008555), *PLAT* (tPA) (QT00075761), *CDH1* (E-cadherin) (QT00080143), or *ACTB* (β-actin) (QT01680476) with QuantiNova SYBR Green RT-PCR kits (Qiagen). Relative transcript levels of target genes were normalized to *ACTB* (β-actin).

### Wound-healing migration assay

The wound-healing migration assays were done in serum-starved fibroblasts. Confluent monolayers of cells were wounded with a p20 pipette tip (time 0). Phase-contrast images were taken using an Olympus inverted microscope at time 0 h or 24 h after the scratch wound. Wound areas were evaluated by ImageJ 1.42q software.

### Transwell migration assay

For the transwell migration assay, transwell membranes (8-μm pore size, 6.5-mm diameter; Corning Costar; 3422) were used (45, 55, 56). The bottom chambers of the transwell were filled with indicated CM. The top chambers were seeded with 1.5 × 10^5^ serum-starved fibroblasts per well. After 24 h, the filters were fixed with 4% paraformaldehyde for 10 min at room temperature; subsequently, the cells on the upper side of the membrane were scraped with a cotton swab. Filters were stained with crystal violet for light microscopy. Images were taken using an Olympus inverted microscope, and migratory cells were evaluated by ImageJ 1.42q software.

### Quantitative proteomic analysis of the fibroblasts secretome and data processing

Proteomic experiment was performed in three biological replicates. Serum-free CM from MRC5 cells treated with or without TGF-β1 (10 ng/ml; 48 h) were enriched based upon Strataclean resin (Agilent) and analyzed using LC–MSE (57), to provide estimates of absolute protein concentration with in-depth coverage. Raw data were analyzed and exported into a csv document. Normalization method for each data is first dividing by the sum of each sample, using proteins containing no missing data, then multiplying by 10,000 (6). Minimum values of each column after normalization were used as pseudocounts to replace all the missing data, allowing for a complete statistical analysis (58). Differentially expressed proteins between two groups with *p*  <  0.05 were identified as significant using a two-tailed and unpaired Student's *t* test (details described in supporting information section).

### Statistical analysis and repeatability of experiments

Each experiment was repeated at least twice. Data are presented as mean and SD and compared with two-sample *t* test, Welch two-sample *t* test, Mann–Whitney *U* test, or one-way ANOVA test if appropriate. *p* < 0.05 was considered statistically significant. All data analyses and graphs were done in RStudio (version 3.6.1) or GraphPad Prism (version 8.2.1, GraphPad Software, Inc). Codes are available upon request.

### Data availability

RNA-Seq data have been deposited in the Gene Expression Omnibus database (accession code: GSE163908). The mass spectrometry proteomics data have been deposited to the ProteomeXchange Consortium *via* the PRIDE repository (59) with the dataset identifier PXD025821.

## Supporting information

This article contains supporting information (57).

## Conflict of interest

The authors declare that they have no conflicts of interest with the contents of this article.
