# Supplementary material for: Bidirectional epithelial–mesenchymal crosstalk provides self-sustaining profibrotic signals in pulmonary fibrosis
Source: J Biol Chem. 2021 Aug 18;297(3):101096. doi: 10.1016/j.jbc.2021.101096 (PMC8435701; doi:10.1016/j.jbc.2021.101096)
Supplement: Figures S1–S6 [file mmc1.pdf]

Supplementary Methods

Quantitative proteomic analysis of the fibroblasts secretome.

1. *Sample preparation.* Serum-free conditioned media (CM) from MRC5 lung fibroblasts treated with or without TGF-β1 (10ng/ml, 48 h) were centrifuged (17,000 *g*, 1 min) and the supernatants were collected (n = 3 biological replicates). 50 μL of the slurry from the pre-vortexed StrataClean resin was added to 1 mL of CM supernatant. Sample mixture was incubated for 1 h at 4°C with vigorous shaking and then centrifuged (17,000 *g*, 3 min) to allow proteins to bind to the resin. Proteins were resuspended in 300 μL 100 mM ammonium bicarbonate buffer containing 0.1% SDS, reduced for 1 hour by 0.5 μg of dithiothreitol (DTT) at 60°C on a thermomixer, alkylated for 45 minutes by 2.5 μg of iodoacetamide at room temperature in the dark and subsequently digested overnight using 0.5μg sequencing grade modified trypsin at 37°C. Digested samples were centrifuged (17,000 *g*, 3 min) and 500 μL of 100% acetonitrile was used to further wash the resin. After centrifuged (17,000 *g*, 3 min), two supernatants were combined and lyophilized at 35°C in a vacuum centrifuge. Lyophilized peptides were reconstituted in 150 μL reconstitution buffer (3% acetonitrile + 0.1% formic acid), vortexed and acidified to pH < 3.0 with TFA. Acidic samples were then loaded onto equilibrated Empore C18 96-well solid phase extraction plates (3M). Each sample was washed with 150 μL of 0.5% acetic acid and eluted by using 200 μL of 80% acetonitrile + 0.5% acetic acid. Each supernatant was lyophilized at 35°C in a vacuum centrifuge and resuspended in 25 μL of loading buffer (2% acetonitrile + 0.1% formic acid) containing 100 fmol/μL of the internal enolase digestion standard (Waters) for further MS analysis.

2. *LC-MS^E analysis.* A reverse-phase trap column (Symmetry C18, 5 μm, 180 μm x 20mm, Waters, Milford, MA) was used for loading the digested samples at a trapping rate of 5 μL/min and washed for 10 min with loading buffer before nanoscale LC separation on a C18 reversed-phase column (HSS T3, 1.8 μm, 500 mm x 75μm, Waters). The peptides were

eluted for 2 hours using 0.1% formic acid accompanying with linear gradient acetonitrile (from 1% to 60 %), at a flow rate of 300 nL/min. Eluted samples were sprayed into an HDMS system operated Synapt G2-Si mass spectrometer (Water, Wilmslow, UK). Data from 50 to 2000 *m/z* were acquired after alternate low (5V) and elevated (15 to 40 V) collision energy (CE) scans. Ion mobility with a wave height of 650 m/s combined with a wave velocity of 40 V was implemented before fragmentation. For correction, Glu[1]-Fibrinopeptide B ((M+2H)+2, *m/z* = 785.8426) with a concentration of 100 fmol/µL and a flow rate of 250 nL/min was used as the lock mass and was acquired every 1 min.

3. *Database searches.* ProteinLynx Global Server 3.0 (Waters) was used to process the raw data. Regression tester, a custom package was executed. Threshold inspector (Waters) was used to determine the applied setting for optimal peak detection across all datasets: high energy = 30 counts. low energy = 100. For database searches, a regression tester was used against the Uniprot human reference database (24/07/2017; 71,599 entries) containing extra sequence information for the internal standard Enolase. For tryptic digestion, 2 missed cleavages were allowed: a fixed cysteine carboxyamidomethylation and a variable methionine oxidation modification. Ion mass tolerances between products and precursors were calculated automatically. False discovery rate (FDR) was estimated with decoy-fusion database searcher. 4% was first set as the threshold for false discovery rate (FDR), and it was then filtered to 1%. The top 3 method (1) was used to estimate the absolute amounts in fmol. Custom Python scripts were used to comply the ion accounting output files, to generate summary data from search log files, and to collate ion accounting files into a single csv file.

Reference:

1. Silva JC, Gorenstein MV, Li G-Z, Vissers JP, Geromanos SJ. Absolute quantification of proteins by LCMSE: a virtue of parallel MS acquisition. *Molecular & Cellular Proteomics* 2006, **5**(1)**:** 144-156.

Supplementary Figure Legends

Supplementary Fig. 1 Global transcriptomic changes in fibroblasts exposed to conditioned media from RAS-activated ATII cells. Extracellular matrix **(**ECM) (**a**) or migration related (**b**) GO terms were visualized on bar charts, showing number of shared genes and $-\text{Log}_{10}$(FDR). ECM: extracellular matrix. GO: gene ontology. FDR: false discovery rate.

Supplementary Fig. 2 RAS-activated ATII cells augment fibroblast migration, but not cell viability, via paracrine signalling. **a** Scratch wound assay of MRC5 lung fibroblasts treated with conditioned media (CM) from control or 4-OHT-activated $\text{ATII}^{\text{ER:KRASV12}}$ cells. Representative images of MRC5 with the indicated treatments at time 0 or 24 hours after the scratch wound. Wounds have been artificially coloured red to aid visualization. Scale bar: 200 μm. Graph showing the areas of wounds evaluated with ImageJ, and data are mean ± s.d. $n = 3$. $^{**}P < 0.01$. *n.s.* (not significant) $P > 0.05$. **b** Graph showing relative cell viability in MRC5 treated with CM from control or 4-OHT-activated $\text{ATII}^{\text{ER:KRASV12}}$ cells using the Cell-Titer Glo® assay. Data are mean ± s.d. $n = 3$. *n.s.* (not significant) $P > 0.05$.

Supplementary Fig. 3 Tissue plasminogen activator (tPA), encoded by gene *PLAT*, is a key paracrine regulator of fibroblast migration. **a** Fold change in mRNA levels of *ZEB1* in $\text{ATII}^{\text{ER:KRASV12}}$ cells with indicated treatment. β-actin-normalized mRNA levels in control cells were used to set the baseline value at unity. Data are mean ± s.d. $n = 3$ samples per group. **b** Scratch wound assay of fibroblasts treated with CM from $\text{ATII}^{\text{ER:KRASV12}}$ cells with indicated treatment. Representative images of MRC5 fibroblasts with the indicated treatment at time 0 or 24 hours after the scratch wound. Wounds have been artificially coloured red to aid visualization. Scale bar: 200 μm. Images for cells treated with the control siRNA are also shown in Fig. S3f. **c** Scratch wound assay of fibroblasts treated with or without recombinant

tPA. Representative images of MRC5 fibroblasts with the indicated treatments at time 0 or 24 hours after the scratch wound. Wounds have been artificially coloured red to aid visualization. Scale bar: 200 μm. Graph shows the areas of wounds evaluated with ImageJ, and data are mean ± s.d. $n = 3$. $^{****}P < 0.0001$. **d** Flow chart showing the experimental procedure for *Supplementary Fig. 3f*. In brief, $ATII^{ER:KRASV12}$ cells were transfected with control (RISC-free) or *PLAT* siRNA (*PLAT* RNAi) for 48 hours. Fresh media were replaced in the presence or absence of 4-OHT (4-hydroxytamoxifen), and conditioned media (CM) were collected after 24 hours. Confluent MRC5 fibroblasts were scratched and treated with indicated CM from ATII cells. **e** Fold change in mRNA levels of *PLAT* in $ATII^{ER:KRASV12}$ cells with the indicated treatment. β-actin-normalized mRNA levels in control cells were used to set the baseline value at unity. Data are mean ± s.d. $n = 3$ samples per group. **f** Scratch wound assay of fibroblasts treated with CM from $ATII^{ER:KRASV12}$ cells with indicated treatment. Representative images of MRC5 fibroblasts with the indicated treatment at time 0 or 24 hours after the scratch wound. Wounds have been artificially coloured red to aid visualization. Scale bar: 200 μm.

Supplementary Fig. 4 TGF-β-activated fibroblasts induce RAS activation in ATII cells. **a** Flow chart showing the experimental procedure for *Supplementary Fig. 4b*. In brief, MRC5 fibroblasts were treated with TGF-β (5 ng/ml) for the indicated duration (24, 48 or 72 hours). For the "48+, 24-" group, MRC5 fibroblasts were first treated with TGF-β (5 ng/ml) for 48 hours followed by replacement with fresh media to remove TGF-β, and cells or conditioned media (CM) were collected after another 24 hours. **b** Protein expression of α-SMA and phospho-Smad2 (p-Smad2) in MRC fibroblasts with indicated treatment. β-tubulin was used as a loading control. **c** Flow chart showing the experimental procedure for *Supplementary Fig. 4d*. In brief, MRC5 fibroblasts were treated with or without 5 ng/ml TGF-β for 48 hours.

Fresh media were replaced to remove TGF-β and CM were collected after 24 hours. ATII cells were then treated with indicated CM from MRC5 for another 24 hours. **d** Protein expression of ZEB1, phospho-AKT (p-AKT), AKT, phospho-ERK (p-ERK) and ERK in ATII cells with the indicated treatment. β-tubulin was used as a loading control. For protein expression of p-ERK, both short and long exposures (respectively) are shown.

Supplementary Fig. 5 SPARC, a TGF-β-induced secreted protein, is highly expressed in IPF. **a** Venn plot showing the identification of TGF-β-induced secrete proteins. In brief, all DEGs (differentially expressed genes) were overlapped with a list of secreted proteins from Human Protein Atlas. **b** Volcano plot showing secreted proteins (predicted) that are differentially expressed in human lung fibroblasts treated with *vs.* without TGF-β (GSE139963). Genes with Log_2[FoldChange] > 1 and FDR (false discovery rate) < 0.05 were considered as DEGs. Red indicates up-regulated and blue down-regulated.

Supplementary Fig. 6 SPARC is a key fibroblast-derived paracrine regulator of RAS activation in ATII cells. **a** Flow chart showing the experimental procedure for *Supplementary Fig. 6b and c*. In brief, NHLFs (normal human lung fibroblasts) were transfected with control (RISC-free) or *SPARC* siRNA (*SPARC* RNAi) for 48 hours, followed TGF-β (5 ng/ml) treatment or not for another 48 hours. Fresh media were replaced to remove TGF-β, and conditioned media (CM) were collected after 24 hours. ATII cells treated with the indicated CM from NHLFs were collected after another 24 hours. **b** Protein expression of SPARC, α-SMA and phospho-Smad2 (p-Smad2) in NHLFs with indicated treatment. β-tubulin was used as a loading control. **c** Protein expression of SPARC in CM from NHLFs with the indicated treatment. Ponceau S staining showing total protein levels. Protein expression of phospho-AKT (p-AKT) and phospho-ERK (p-ERK) in ATII cells treated with indicated CM from

NHLFs. β-tubulin was used as a loading control. **d** Protein expression of SPARC in CM from NHLFs or IPFFs (IPF fibroblasts) with the indicated treatment. Ponceau S staining showing total protein levels. Protein expression of phospho-AKT (p-AKT) and phospho-ERK (p-ERK) in ATII cells treated with CM from NHLFs or IPFFs with indicated treatment. β-tubulin was used as a loading control. **e** Fold change in mRNA levels of *CDH1* (E-cadherin) in ATII cells treated with or without hrSPARC (human recombinant SPARC). β-actin-normalized mRNA levels in control cells were used to set the baseline value at unity. Data are mean ± s.d. n = 3 samples per group. $^{**}P < 0.01$.

Supplementary Figure 1

a

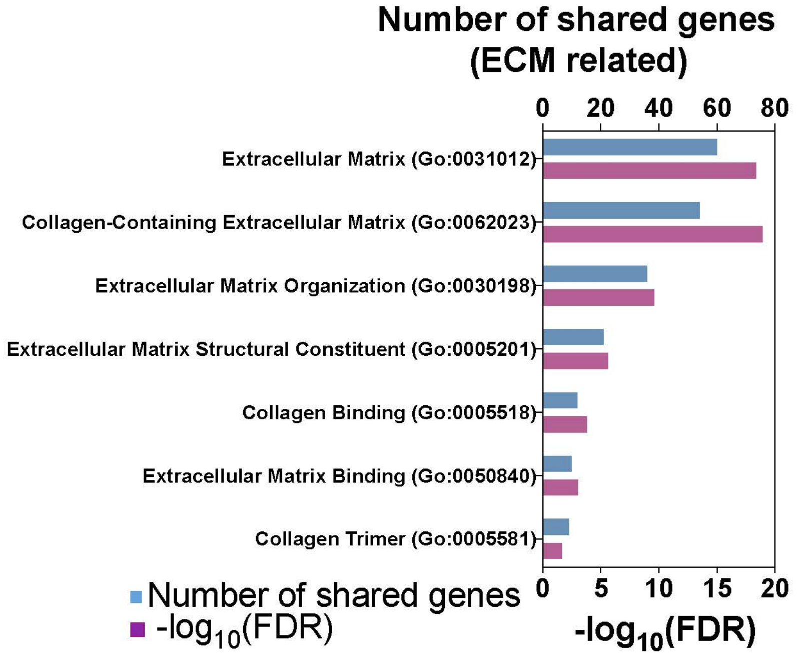

b

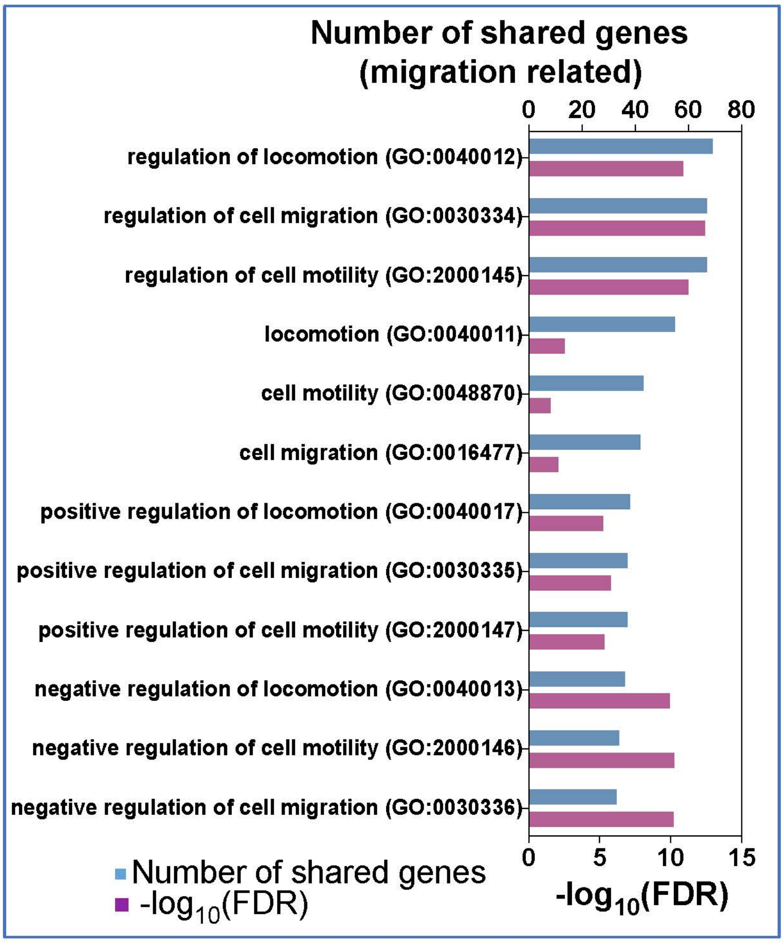

Supplementary Figure 2

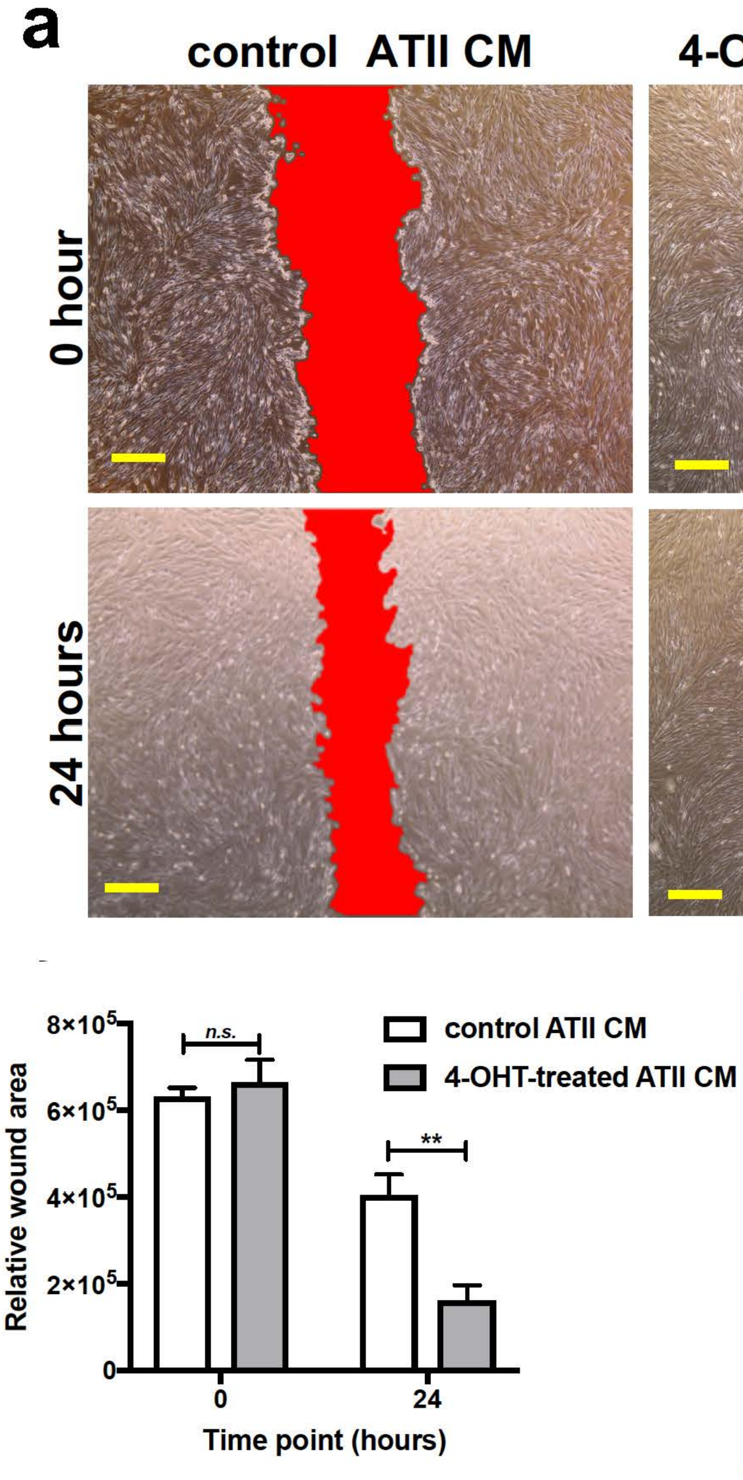

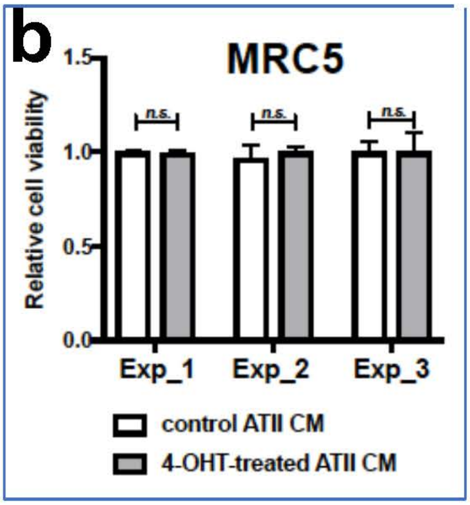

Supplementary Figure 3

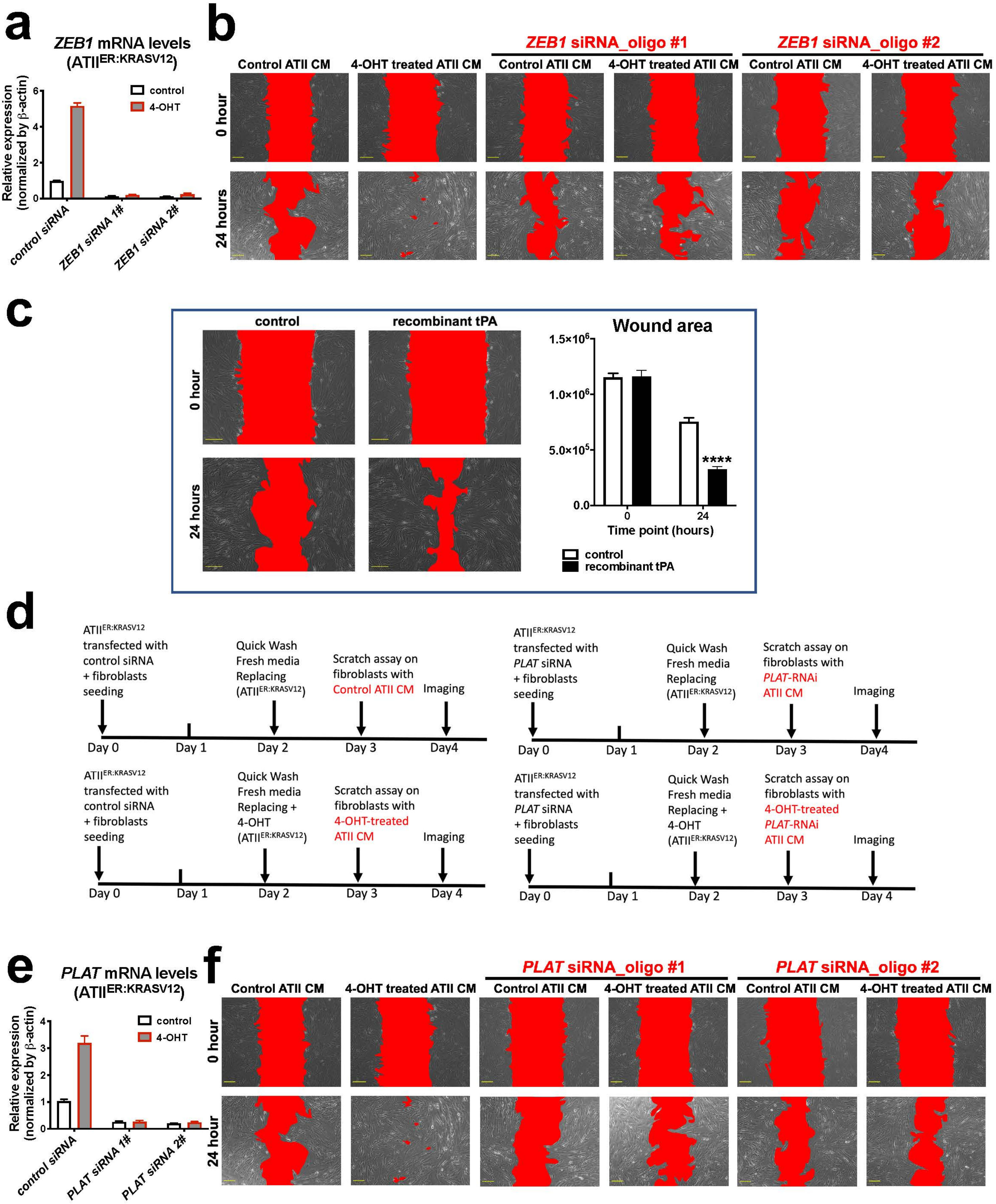

Supplementary Figure 4

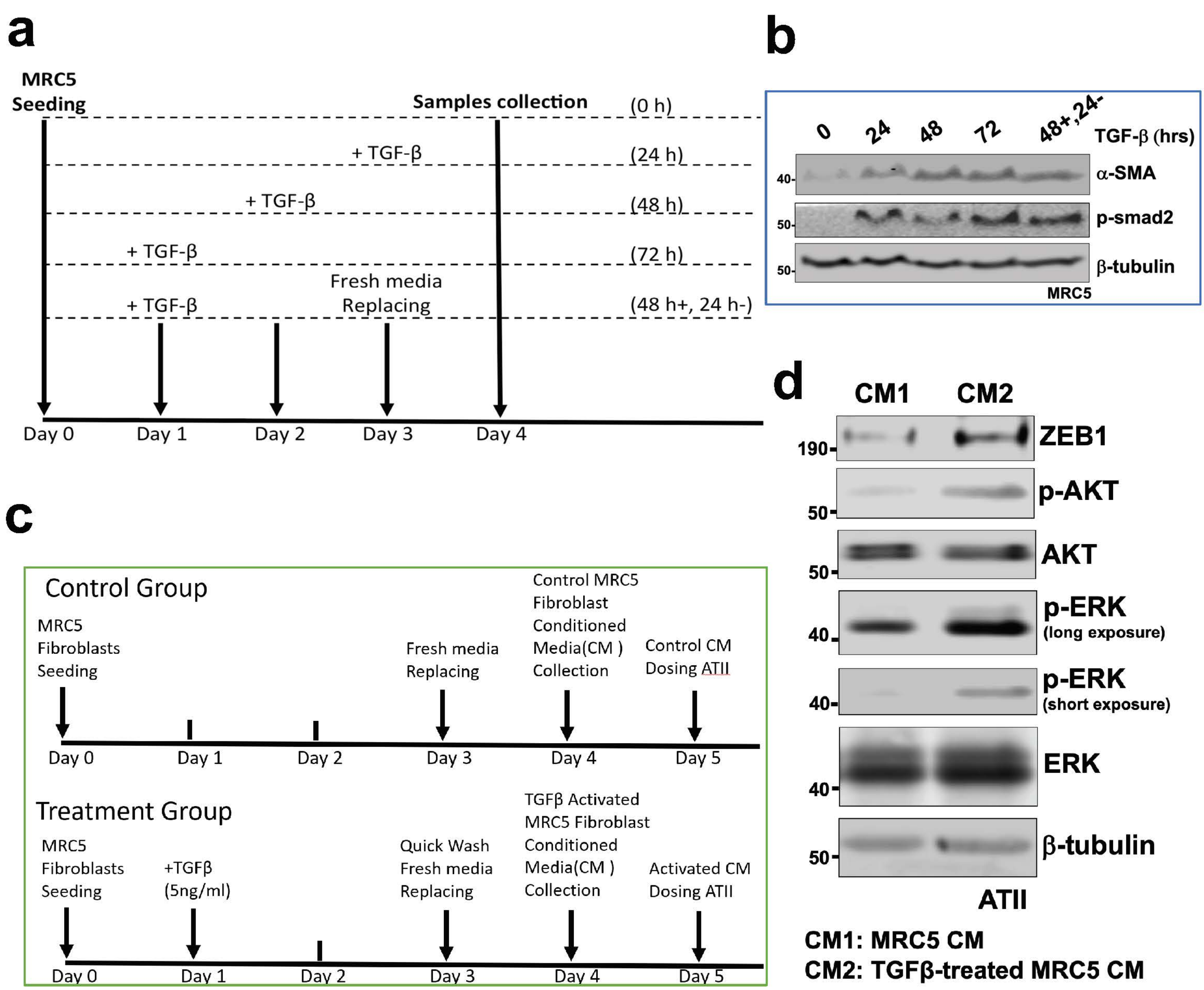

Supplementary Figure 5

a

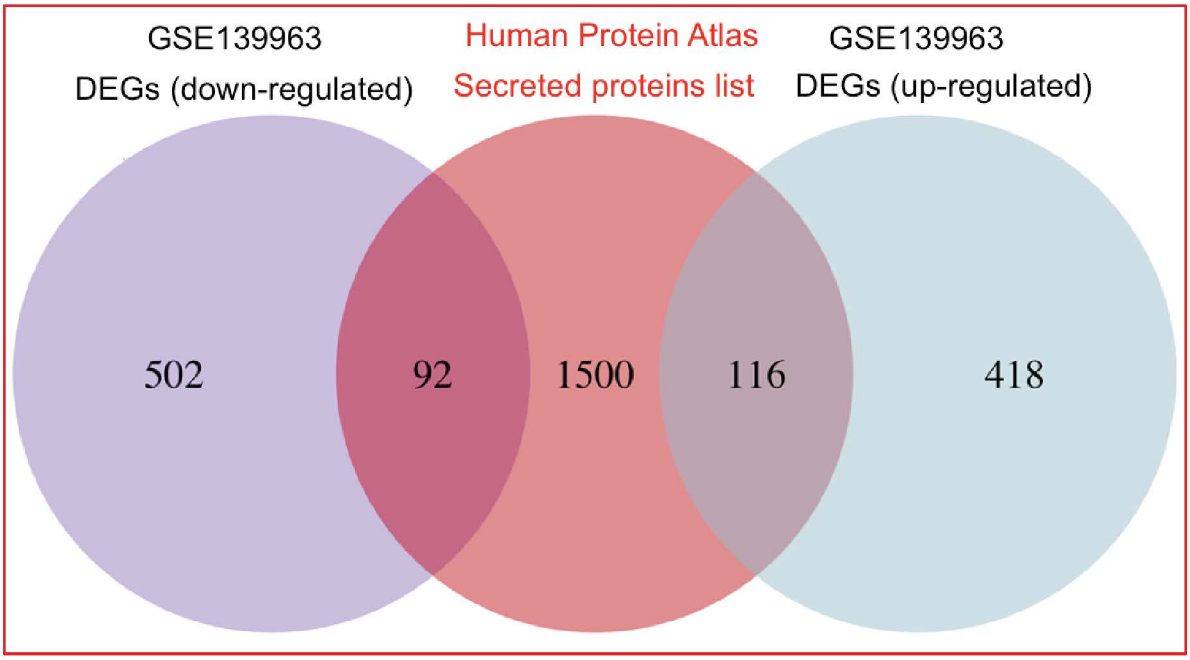

b

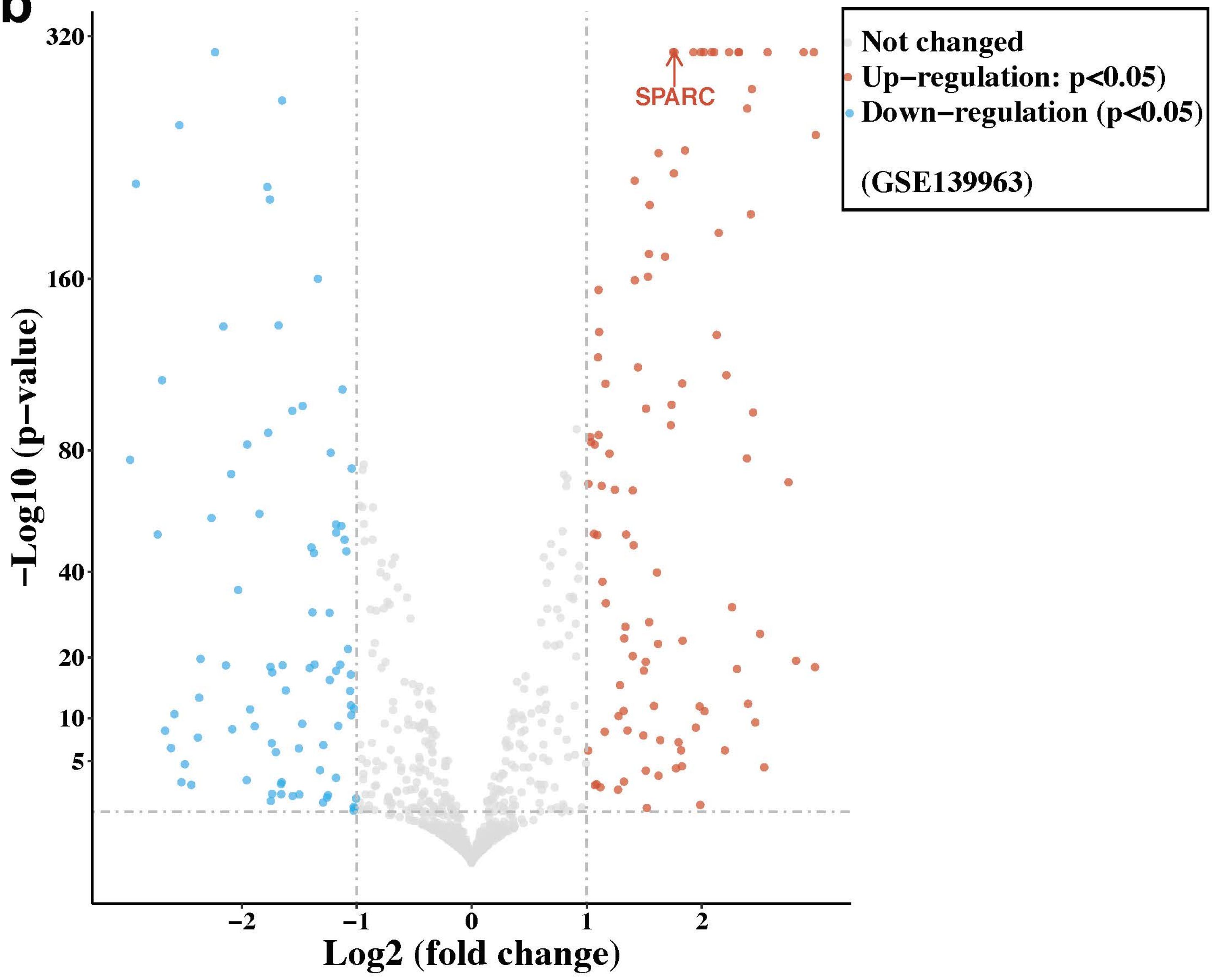

Supplementary Figure 6

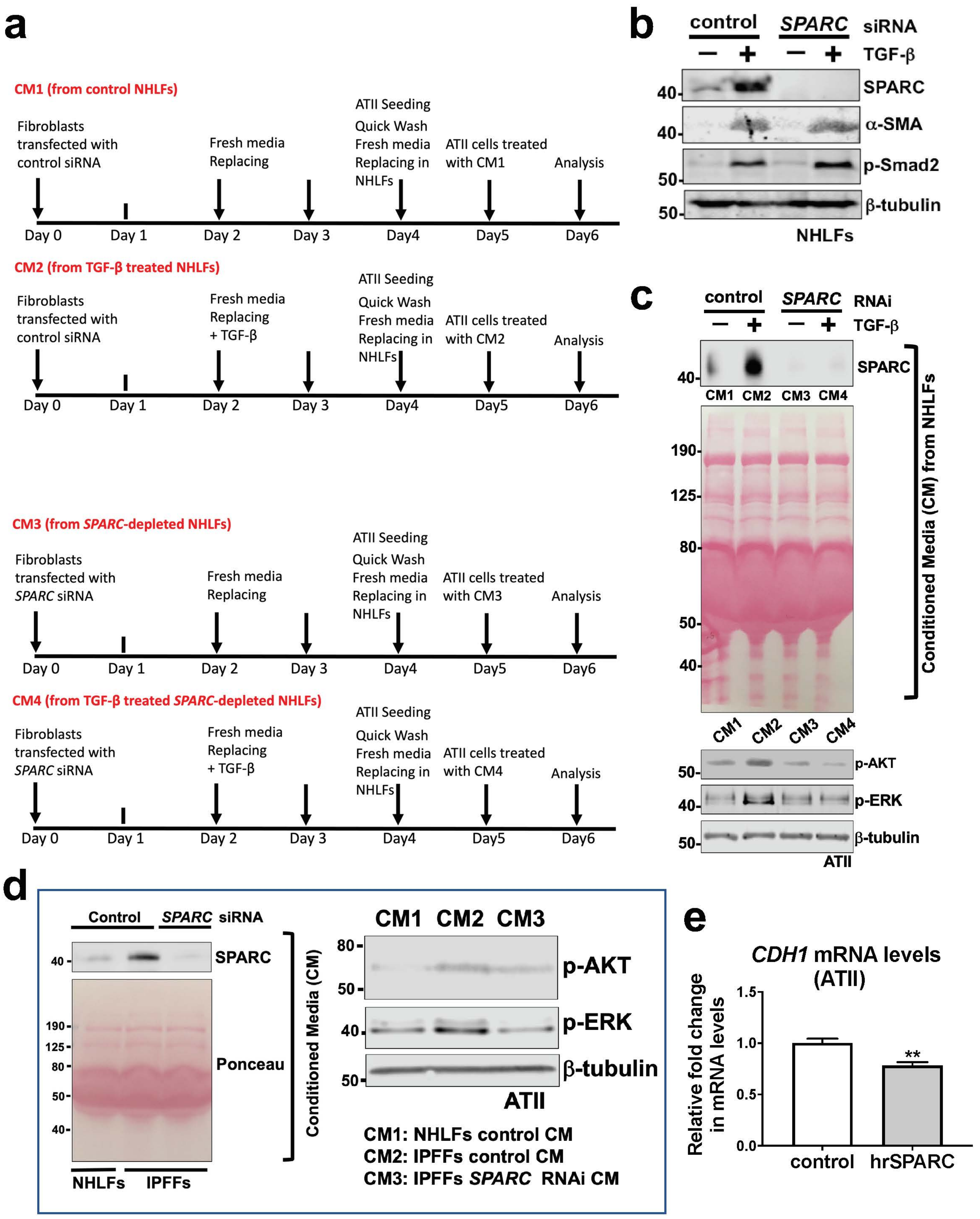

Supplementary Tables

Supplementary Table 1 Up-regulated DEGs in MRC5 cells treated with CM from 4-OHT-treated *vs.* control $ATII^{ER:KRASV12}$ cells.

Supplementary Table 2 Down-regulated DEGs in MRC5 cells treated with CM from 4-OHT-treated *vs.* control $ATII^{ER:KRASV12}$ cells.

Supplementary Table 3 GO analysis of up-regulated DEGs in MRC5 cells treated with CM from 4-OHT-treated *vs.* control $ATII^{ER:KRASV12}$ cells.

Supplementary Table 4 GO analysis of down-regulated DEGs in MRC5 cells treated with CM from 4-OHT-treated *vs.* control $ATII^{ER:KRASV12}$ cells.

Supplementary Table 5 List of secreted proteins in the conditioned media from TGF-β treated (T) *vs.* control (C) MRC5 cells identified by quantitative proteomic analysis.

Supplementary Table 6 Quantitative proteomic analysis in the conditioned media from TGF-β treated *vs.* control MRC5 cells.

Supplementary Table 7 List of up-regulated genes in human lung fibroblasts treated with *vs.* without TGF-β (GSE139963).
